# Effectiveness of AI-assisted rehabilitation for musculoskeletal disorders: a network meta-analysis of pain, range of motion, and functional outcomes

**DOI:** 10.3389/fbioe.2025.1660524

**Published:** 2025-10-16

**Authors:** Zixuan Luo, Yang Wang, Tongyan Zhang, Jiale Wang

**Affiliations:** ^1^ Faculty of Physical Culture, Tomsk State University, Tomsk, Russia; ^2^ School of Sports Science, Xinjiang Normal University, Ürümqi, China

**Keywords:** artificial intelligence, AI-assisted rehabilitation, musculoskeletal disorders, network meta-analysis, robotic rehabilitation, functional recovery, personalized rehabilitation

## Abstract

**Objective:**

This study aims to compare the effectiveness of 13 artificial intelligence (AI)-assisted rehabilitation strategies for individuals with musculoskeletal disorders (MSDs), categorized based on different intervention types, including AI feedback systems, exergaming platforms, telerehabilitation, and robotic solutions. The analysis focuses on improvements in pain relief, functional outcomes, and range of motion (ROM), based on a network meta-analysis (NMA) of randomized controlled trials (RCTs).

**Methods:**

A systematic review and NMA were conducted in accordance with PRISMA guidelines. Four databases (PubMed, Embase, Cochrane Library, Web of Science) were searched for RCTs published between January 2000 and April 2025. A total of 33 RCTs involving participants with MSDs were included. Interventions were categorized into 13 AI-assisted rehabilitation strategies. The outcomes were grouped into three domains: pain, functional outcomes, and ROM. Surface under the cumulative ranking curve (SUCRA) values and mean ranks were used to compare the relative effectiveness of each intervention. The Risk of Bias (RoB 2) tool was used to assess the bias risk of the studies, and the Confidence in Network Meta-Analysis (CINeMA) tool was applied to evaluate the credibility of the evidence.

**Results:**

For pain relief, Therapeutic Exergaming (SUCRA = 87.6%) and Robotic Exoskeleton (SUCRA = 86.3%) ranked highest. In functional outcomes, Gamified Exergaming (SUCRA = 99.6%) and Hybrid Physical Therapy combined with Exergaming (SUCRA = 81.2%) showed superior results. For ROM, Single-Joint Rehab Robot (SUCRA = 84.7%) and AI-Feedback Motion Training (SUCRA = 83.7%) were most effective. Conventional or Usual Care and Asynchronous Telerehabilitation consistently ranked lower across all outcomes.

**Conclusion:**

This study demonstrates that AI-assisted rehabilitation strategies significantly improve pain relief, functional recovery, and ROM in individuals with MSDs. Interventions such as Therapeutic Exergaming, Robotic Exoskeletons, Gamified Exergaming, and Single-Joint Rehab Robots performed excellently in their respective domains, highlighting the potential of AI technologies in personalized treatment and enhancing patient recovery. However, further long-term research is needed to confirm the sustained effects of these interventions and optimize their clinical application.

**Systematic Review Registration:**

PROSPERO CRD420251057777.

## 1 Introduction

Musculoskeletal disorders (MSDs), such as osteoarthritis, tendinopathies, and postoperative impairments, are among the leading causes of disability worldwide (Wang et al., 2024). These conditions not only significantly hinder individuals’ ability to perform daily activities but are also closely linked to chronic pain, reduced quality of life, and escalating healthcare costs (Gaskin and Richard, 2012). Rehabilitation is a key component in managing MSDs, aiming to alleviate pain, restore functional outcomes, and improve range of motion (ROM) (Alaca et al., 2025). However, traditional rehabilitation methods often face challenges, such as varying patient adherence, lack of personalization, delayed feedback mechanisms, and high resource demands (Ntantos et al., 2020).

In recent years, artificial intelligence (AI) has emerged as a transformative force in rehabilitation medicine (Topol, 2019). AI technologies harness advanced algorithms to process complex physiological, biomechanical, and behavioral data, providing real-time, individualized feedback, dynamically adjusting training intensity, and customizing therapy plans based on patient-specific progress (Davenport and Kalakota, 2019). These innovations have led to the development of a wide range of AI-assisted rehabilitation strategies, including AI-driven prescription platforms, motion-feedback systems, robotic exoskeletons, virtual reality (VR)-enhanced therapies, and technology-supported telerehabilitation programs (Louie and Eng, 2016).

The rapid expansion of digital health infrastructure, combined with a growing focus on remote, data-driven, and patient-centered care, has facilitated the clinical integration of intelligent rehabilitation technologies (Meskó et al., 2017). These systems are increasingly used to complement traditional physical therapy or function as independent interventions in outpatient and home settings (Delnevo et al., 2021). With their potential to enhance clinical outcomes, improve patient engagement, and increase access to rehabilitation services, AI-assisted rehabilitation approaches are becoming a key area of research (Lanotte et al., 2023). However, despite the growing body of randomized controlled trials (RCTs) evaluating individual AI-based interventions, there remains a significant gap in comprehensive, comparative evidence that synthesizes their effectiveness across key rehabilitation outcomes—namely pain relief, functional recovery, and ROM improvement (Kapil et al., 2025). This evidence gap hinders clinical decision-making and impedes the optimal implementation of the most effective interventions.

Network meta-analysis (NMA) provides a robust framework to address this gap by enabling simultaneous comparisons of multiple interventions, incorporating both direct and indirect evidence (Jiang et al., 2025). Due to the diversity and complexity of AI-assisted approaches, NMA is particularly well-suited for evaluating their relative performance. The present study, therefore, seeks to conduct a systematic review and NMA of RCTs evaluating 13 distinct AI-assisted rehabilitation strategies for individuals with MSDs. Focusing on three key outcomes—pain relief, functional recovery, and ROM—this study aims to identify the most effective interventions and contribute to the growing evidence supporting intelligent, outcome-driven rehabilitation practices.

## 2 Methods

### 2.1 Study protocol and reporting standards

This systematic review and NMA was conducted in accordance with the Preferred Reporting Items for Systematic Reviews and Meta-Analyses (PRISMA) 2020 guidelines (Page et al.). The PRISMA checklist is available in the supplementary materials. The review protocol was prospectively registered in the International Prospective Register of Systematic Reviews (PROSPERO) under registration number CRD420251057777.

### 2.2 Data sources and search strategy

A comprehensive literature search was performed across four electronic databases: PubMed, Embase, the Cochrane Library, and Web of Science (Bramer et al., 2017). The search covered publications from January 2000 to April 2025 and targeted RCTs evaluating AI-assisted rehabilitation for MSDs.

The strategy combined both keywords and Medical Subject Headings (MeSH) related to MSDs, AI, rehabilitation, and randomized trials, using Boolean operators (AND, OR). No language restrictions were applied. The complete PubMed search syntax is provided in Supplementary Table S1.

### 2.3 Study selection

Studies were selected according to the PICOS framework: Population (P): adults with musculoskeletal disorders (MSDs), including osteoarthritis, tendinopathies, ligament or tendon injuries, postoperative rehabilitation, and chronic musculoskeletal pain; Interventions (I): AI-assisted rehabilitation strategies classified into 13 types (e.g., AI-feedback motion training, AI-prescription apps, telerehabilitation, VR-based therapies, exergaming, robotic systems, multimodal platforms); Comparators (C): conventional or usual care, or other AI/digital interventions; Outcomes (O): pain, functional outcomes, and range of motion (ROM) measured with validated tools (e.g., VAS, WOMAC-Function, KOOS-ADL, goniometry); Study design (S): randomized controlled trials. Exclusion criteria were non-randomized designs, absence of AI-assisted components, or lack of relevant outcome data.

### 2.4 Data extraction

Two reviewers independently extracted the following information from each eligible study: first author and year of publication, country, study design and sample size, participant characteristics, intervention and control details, duration of intervention, and outcome measures. Discrepancies were resolved through discussion or consultation with a third reviewer (Büchter et al., 2020). In addition to these study-level variables, we also extracted detailed rehabilitation protocol characteristics (e.g., intervention frequency, session duration, supervision, setting, and exercise type) to allow meaningful comparison across trials.

### 2.5 Classification and characteristics of 13 AI-assisted rehabilitation interventions

AI-assisted rehabilitation encompasses a wide range of approaches with different technological foundations, delivery formats, and therapeutic objectives. To ensure systematic comparison and consistency with our subsequent network meta-analysis (NMA), all included interventions were categorized into 13 distinct types, spanning AI-based systems, robotics, virtual reality (VR), telerehabilitation, exergaming, and multimodal platforms (The detailed classification is summarized in Table 1).

**TABLE 1:** Classification of AI-Assisted rehabilitation interventions.

Intervention type	Definition (with representative references)	Main features	Intended rehabilitation goals
AI-Feedback Motion Training	Uses sensors and AI algorithms to analyze motion and provide real-time feedback (Topol, 2019; Sánchez-Gil et al., 2025)	Corrects posture, personalized feedback	Improve movement accuracy, reduce compensations
AI-Prescription App	AI-powered app generates individualized rehab plans from patient data (Lanotte et al., 2023; Alshami et al., 2025; Jin et al., 2018)	Dynamic plan adjustment, self-management	Personalize therapy, optimize adherence
Asynchronous Telerehabilitation	Patients follow pre-set rehab plans independently, feedback provided later (Rasa, 2024; Abedi et al., 2024; Collado-Mateo et al., 2017)	High flexibility, no real-time supervision	Enable home-based rehab, reduce access barriers
Synchronous Telerehabilitation	Real-time remote rehab guided by therapist (Alfieri et al., 2022; Timurtas et al., 2023; Azma et al., 2018)	Live interaction, immediate adjustments	Enhance adherence, ensure correct performance
Gamified Exergaming	Exercise integrated with game mechanics (scores, rewards) (Timurtaş et al., 2025; Anan et al., 2021)	Fun, motivational elements	Increase engagement, improve compliance
Therapeutic Exergaming	Clinically designed game-based tasks supervised by professionals (Rasa, 2024; Maeda et al., 2024; Marcuzzi et al., 2023)	Goal-oriented, therapeutic alignment	Achieve specific functional outcomes
Feedback VR Platform	VR with integrated motion feedback (Davenport and Kalakota, 2019; Jiang et al., 2025; Dahl-Popolizio et al., 2014)	Immersive training + corrective feedback	Improve sensorimotor control, functional recovery
Immersive VR System	Fully immersive VR world for training (Davenport and Kalakota, 2019; Louie and Eng, 2016; Bossen et al., 2013; Cetin et al., 2022)	High ecological validity, intensive training	Enhance balance, mobility, cognitive-motor integration
Multimodule Digital App	Digital app integrating AI, monitoring, and feedback (Kapil et al., 2025; Abedi et al., 2024; Jin et al., 2018)	Comprehensive management (exercise, pain, tracking)	Support holistic recovery, promote adherence
Multimodal Digital Platform	Combines AI, VR, robotics in one platform (Delnevo et al., 2021; Jiang et al., 2025; Hardt et al., 2018)	Multidimensional therapeutic support	Address physical, psychological, functional needs
Robotic Exoskeleton	Wearable robotic device assisting locomotion/upper limb (Topol, 2019; Louie and Eng, 2016; Bäcker et al., 2021)	Provides mechanical support	Restore walking ability, mobility in severe impairments
Single-Joint Rehab Robot	Robotic device targeting a specific joint (Topol, 2019; Davenport and Kalakota, 2019; Ditch et al., 2020)	High-precision repetitive training	Regain joint function, increase ROM
Hybrid PT + Exergaming	Conventional physiotherapy combined with exergames (Alfieri et al., 2022; Timurtas et al., 2023; Anan et al., 2021)	Blends clinical rigor with engagement	Improve clinical outcomes while maintaining motivation

As summarized in Table 1, AI-assisted rehabilitation interventions can be categorized according to their underlying technologies and therapeutic objectives. AI-based systems, such as AI-feedback training and prescription apps, focus on real-time monitoring and dynamic personalization; robotics, including exoskeletons and single-joint rehabilitation robots, deliver mechanical assistance for patients with severe impairments; VR-based approaches, whether feedback- or immersion-oriented, provide engaging virtual environments to facilitate motor recovery and balance; telerehabilitation, in synchronous or asynchronous formats, enhances accessibility while varying in therapist involvement; exergaming, which may be gamified or therapeutic, integrates exercise with motivational or clinically designed game tasks; and multimodal platforms combine several technologies, such as AI, VR, and robotics, to offer comprehensive rehabilitation solutions. This classification framework forms the analytical foundation of our NMA, enabling structured comparisons across diverse AI-assisted strategies and their relative effects on pain, range of motion, and functional outcomes.

### 2.6 Risk of bias assessment

The risk of bias for each included study was assessed using the Cochrane RoB 2 tool (Delgado et al., 2018). Seven domains were evaluated: (1) Random sequence generation (selection bias), (2) Allocation concealment (selection bias), (3) Blinding of participants and personnel (performance bias), (4) Blinding of outcome assessment (detection bias), (5) Incomplete outcome data (attrition bias), (6) Selective reporting (reporting bias), and (7) Other bias. Risk of bias judgments were categorized as “low risk,” “some concerns,” or “high risk” in accordance with the RoB 2 manual. Any discrepancies between reviewers were resolved by discussion and consensus. Additionally, the CINeMA tool was used to assess the credibility of each comparison, evaluating factors such as imprecision, heterogeneity, and indirectness. This tool helped determine the quality of evidence for each comparison based on a systematic evaluation of these factors.

### 2.7 Outcomes

The primary outcomes of interest were classified into three domains:

1. Pain–measured using the Visual Analog Scale (VAS), Numerical Rating Scale (NRS), or comparable tools (Delgado et al., 2018);
2. Functional outcomes–assessed using validated scales such as the KOOS–Activities of Daily Living (KOOS–ADL) and the WOMAC–Function subscale (Roos and Lohmander, 2003; Bellamy et al., 1988);
3. ROM–evaluated using goniometry or other clinically accepted measurement instruments (Hanks and Myers, 2023).

If multiple assessment tools were reported for the same domain, preference was given to widely validated and frequently applied instruments (e.g., VAS or NRS for pain; KOOS–ADL or WOMAC–Function for functional outcomes). When different tools were used across studies within the same outcome domain, their scores were harmonized by converting them into standardized mean differences (SMDs). This approach ensured that results derived from heterogeneous instruments could be pooled and compared on a common scale.

### 2.8 Statistical analysis

The NMA was conducted using Stata version 15.1 (StataCorp, College Station, TX), applying a random-effects model to account for between-study heterogeneity. Treatment effects were ranked based on the surface under the cumulative ranking curve (SUCRA), mean rank, and the probability of being the best treatment (PrBest) (Rücker and Schwarzer, 2015). Consistency between direct and indirect evidence was assessed using both the design-by-treatment interaction model and node-splitting analyses (Dias et al., 2013). Network plots were generated to illustrate the structure of treatment comparisons. Potential publication bias was evaluated through comparison-adjusted funnel plots, used to visually assess the presence of small-study effects (Chaimani and Salanti, 2012). All statistical analyses were performed separately for each of the three outcome domains: pain, functional outcomes, and ROM.

Subgroup analyses were additionally conducted according to participant characteristics (mean age <60 vs. ≥60 years), baseline disease severity (mild–moderate vs. moderate–severe musculoskeletal disorders), and clinical condition (acute postoperative vs. chronic musculoskeletal pain conditions, including chronic low back pain, chronic neck pain, knee osteoarthritis, and fibromyalgia).

## 3 Results

### 3.1 Study selection

**FIGURE 1:**
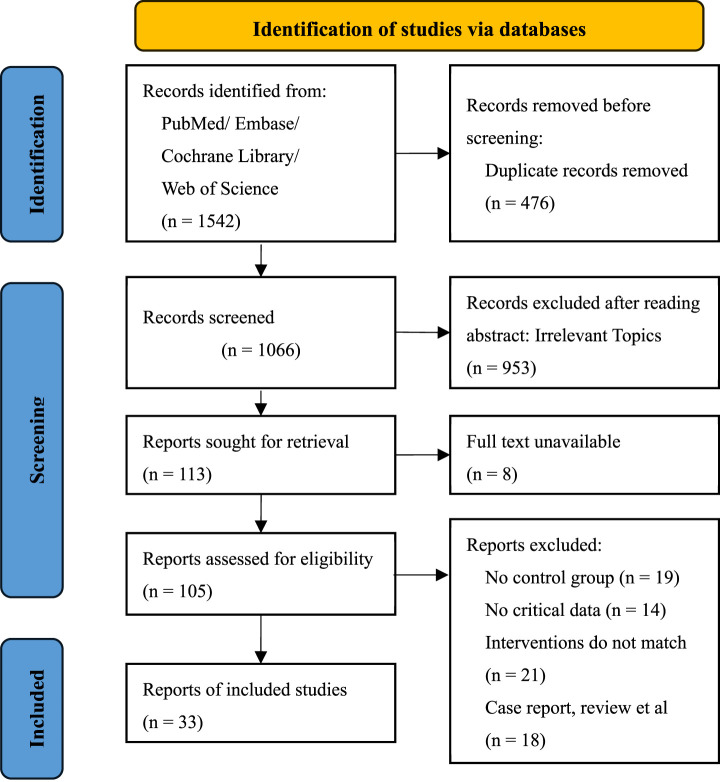
PRISMA flow diagram of study selection.

A total of 1,542 records were retrieved from PubMed, Embase, Cochrane Library, and Web of Science. After removing 476 duplicates, 1,066 records were screened by title and abstract. Of these, 953 were excluded for irrelevance. Among the 113 full-text articles assessed for eligibility, 8 were unavailable. Of the remaining 105 studies, 72 were excluded due to lack of a control group, missing outcome data, unmatched interventions, or non-RCT design. Ultimately, 33 RCTs were included in the final analysis. The detailed selection process is illustrated in the PRISMA flow diagram (Figure 1).

### 3.2 Study characteristics

A total of 33 RCTs, conducted between 2013 and 2024 across 15 countries, were included in this review (Albanese et al., 2021; Allen et al., 2018; Anan et al., 2021; Azma et al., 2018; Bäcker et al., 2021; Bini and Mahajan, 2017; Bossen et al., 2013; Cetin et al., 2022; Collado-Mateo et al., 2017; Dahl-Popolizio et al., 2014; Ditch et al., 2020; Hardt et al., 2018; I et al., 2019; Jin et al., 2018; Kim et al., 2014; Kotani et al., 2020; Maeda et al., 2024; Marcuzzi et al., 2023; Mete and Sari, 2022; Piqueras et al., 2013; Prabhu et al., 2020; Rini et al., 2015; Sarig et al., 2018; Sarig Bahat et al., 2015; Tanaka et al., 2017; Toelle et al., 2019; Tripuraneni et al., 2021; Yoon and Son, 2020; Yu et al., 2023; Zadro et al., 2019; Zhang et al., 2024; Timmers et al., 2019; Mehrholz et al., 2020). All studies involved participants with MSDs and compared AI-assisted rehabilitation strategies with conventional or usual care. Sample sizes ranged from 8 to 327 participants. The interventions were categorized into 13 predefined AI-assisted types. The duration and frequency of interventions varied, from single-session treatments to 12-month rehabilitation programs. Reported outcomes included pain, functional outcomes, and ROM, with many studies contributing data to multiple outcome domains. A summary of the study characteristics is provided in Supplementary Table S2. In addition to the classification of interventions, we summarized the rehabilitation protocols across included RCTs (Supplementary Table S2a), highlighting variations in frequency, session duration, supervision, setting, and exercise type, which are critical for interpreting clinical outcomes.

### 3.3 Risk of bias assessment summary

The risk of bias for all included studies was assessed using the Cochrane RoB 2 tool, with the results summarized in Supplementary Figure S1. Most studies were rated as having low risk or some concerns, with “deviations from intended interventions” identified as the most common high-risk domain. Additionally, the CINeMA tool was used to assess the credibility of each comparison. Most comparisons were rated with high confidence; however, issues such as heterogeneity and inconsistency were common concerns, leading to lower confidence ratings for some comparisons. For further details on the comparisons and credibility ratings, please refer to Supplementary Tables S3–S5.

### 3.4 Network meta-analysis

NMA were conducted separately for each of the three primary outcome domains: pain, functional outcomes, and ROM. Consistency between direct and indirect evidence was assessed using node-splitting analysis, and treatment rankings were evaluated based on SUCRA values. The results for each outcome domain are reported in the following subsections. For clarity in network plots and statistical analyses, each intervention type was assigned a standardized abbreviation (e.g., AI-App = AI-Prescription App, Control = Conventional or Usual Care). A complete list of intervention categories and their corresponding abbreviations is provided in Supplementary Table S6.

#### 3.4.1 Pain outcomes

**FIGURE 2:**
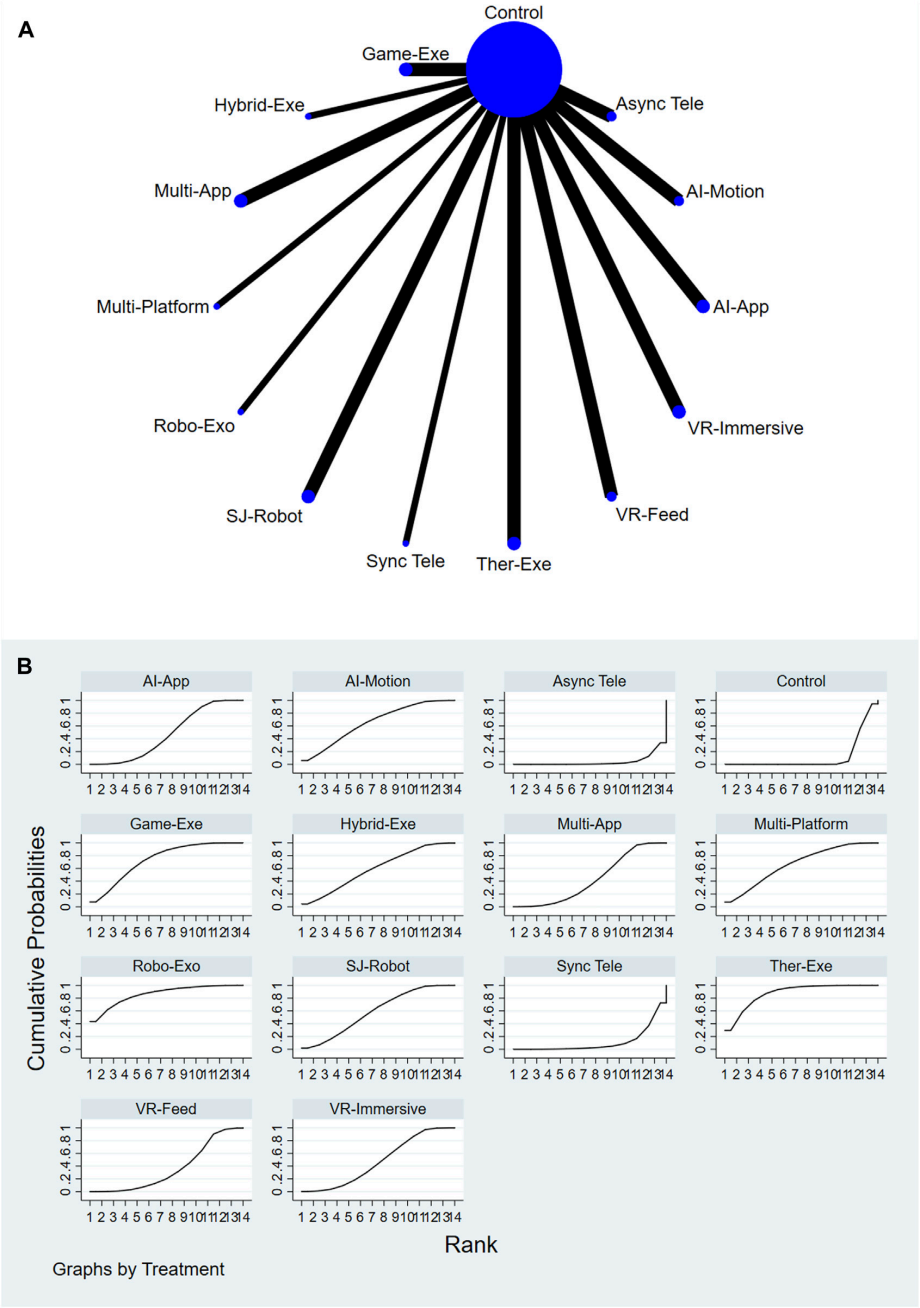
**(A)** Network plot of pain comparisons. **(B)** SUCRA ranking of interventions for pain.

A total of 13 AI-assisted interventions and a control group were evaluated for their effectiveness in reducing pain. Figures 2A,B illustrate the network structure and the corresponding SUCRA-based rankings. The top-ranked interventions included Therapeutic Exergaming (SUCRA = 87.6%), Robotic Exoskeleton (SUCRA = 86.3%), and Gamified Exergaming (SUCRA = 73.7%). In contrast, Asynchronous Telerehabilitation (SUCRA = 4.2%) and Conventional or Usual Care (SUCRA = 12.0%) consistently ranked among the lowest across all comparisons. For a comprehensive summary of SUCRA rankings across all interventions and outcomes, please refer to Supplementary Table S11.

Relative treatment effects are summarized in the league table (see Supplementary Figure S2). Interventions such as Therapeutic Exergaming, Robotic Exoskeleton, Feedback VR Platform, and Gamified Exergaming demonstrated more favorable performance compared to Conventional or Usual Care and other comparators. Node-splitting analysis revealed no significant inconsistency between direct and indirect evidence (P > 0.05), and detailed results are provided in Supplementary Table S7.

#### 3.4.2 Functional outcomes

**FIGURE 3:**
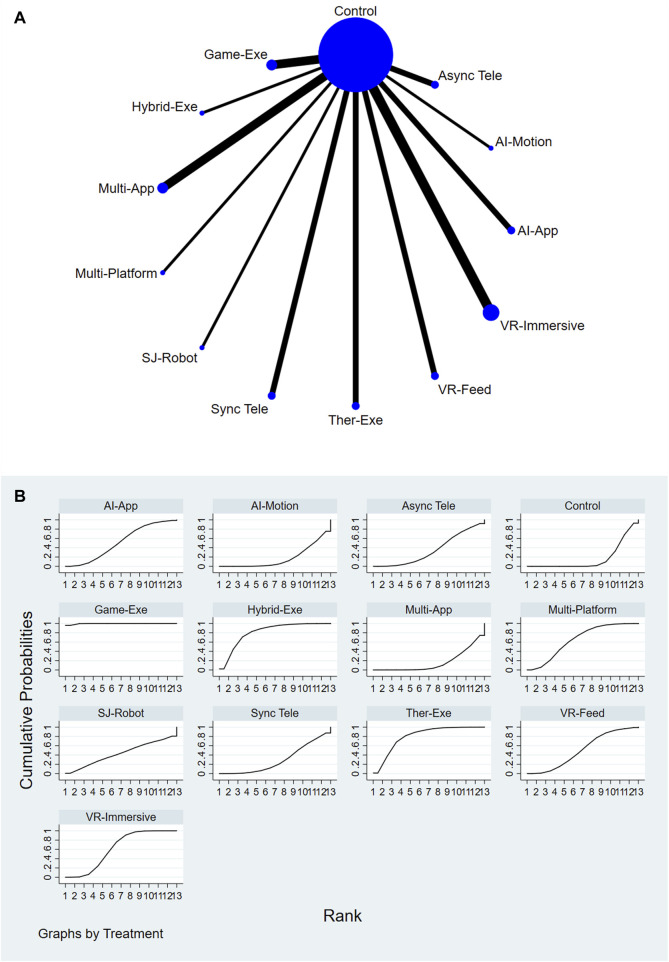
**(A)** Network plot of functional outcomes comparisons. **(B)** SUCRA ranking of interventions for functional outcomes.

A total of 12 AI-assisted interventions and a control group were assessed for their effectiveness in improving functional outcomes. Figures 3A,B illustrate the network structure and corresponding SUCRA rankings. The highest-ranked interventions included Gamified Exergaming (SUCRA = 99.6%), Hybrid Physical Therapy combined with Exergaming (SUCRA = 81.2%), and Therapeutic Exergaming (SUCRA = 80.4%). In contrast, Conventional or Usual Care (SUCRA = 17.1%) and AI-Feedback Motion Training (SUCRA = 17.8%) consistently ranked among the lowest across all comparisons. For a comprehensive summary of SUCRA rankings across all interventions and outcomes, please refer to Supplementary Table S11.

Relative treatment effects are presented in the league table (see Supplementary Figure S3). Interventions such as Gamified Exergaming and Therapeutic Exergaming demonstrated consistently favorable performance across the network. Node-splitting analysis revealed no significant inconsistency between direct and indirect evidence (P > 0.05); detailed results are provided in Supplementary Table S8.

#### 3.4.3 ROM outcomes

**FIGURE 4:**
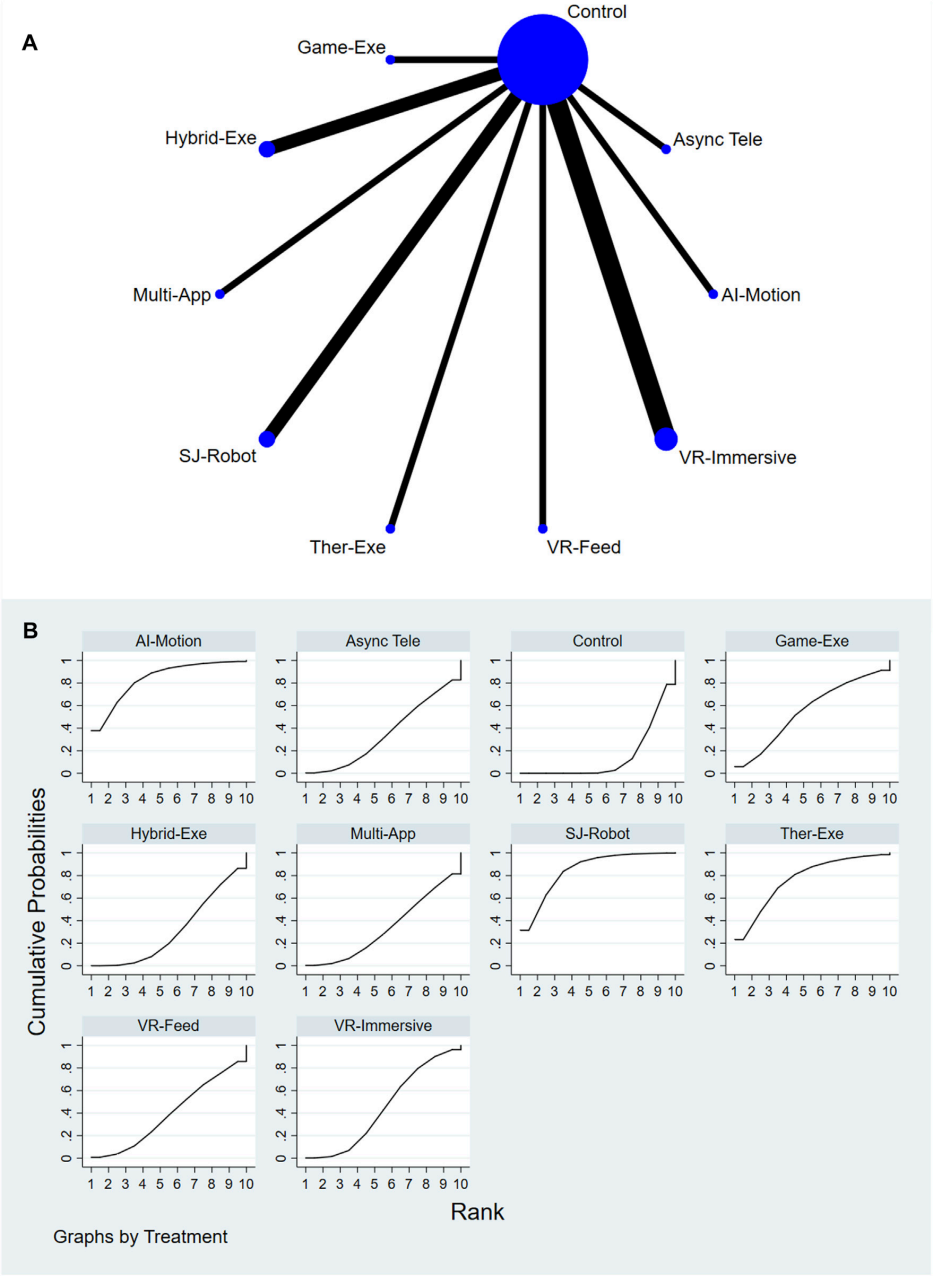
**(A)** Network plot of ROM comparisons. **(B)** SUCRA ranking of interventions for ROM.

A total of 9 AI-assisted interventions and a control group were assessed for their effects on ROM. Figures 4A,B present the network structure and the corresponding SUCRA rankings. The top-performing interventions were Single-Joint Rehab Robot (SUCRA = 84.7%), AI-Feedback Motion Training (SUCRA = 83.7%), and Therapeutic Exergaming (SUCRA = 76.8%). In contrast, Conventional or Usual Care (SUCRA = 15.0%) and Gamified Exergaming (SUCRA = 31.2%) consistently ranked among the lowest across all comparisons. For a comprehensive summary of SUCRA rankings across all interventions and outcomes, please refer to Supplementary Table S11.

Relative treatment effects are presented in the league table (see Supplementary Figure S4). Interventions such as AI-Feedback Motion Training, Therapeutic Exergaming, and Single-Joint Rehab Robot demonstrated greater improvements in ROM compared to other interventions. Node-splitting analysis revealed no significant inconsistency between direct and indirect evidence (P > 0.05); detailed results are provided in Supplementary Table S9.

Subgroup analyses are summarized in Supplementary Table S10, which presents pooled effect sizes, heterogeneity estimates, and statistical significance across subgroups. The results suggest that younger patients and those with mild-to-moderate musculoskeletal disorders benefited more, while acute postoperative populations showed more pronounced short-term improvements, although evidence on long-term effects in elderly or more severe cases remains limited.

### 3.5 Assessment of publication bias

**FIGURE 5:**
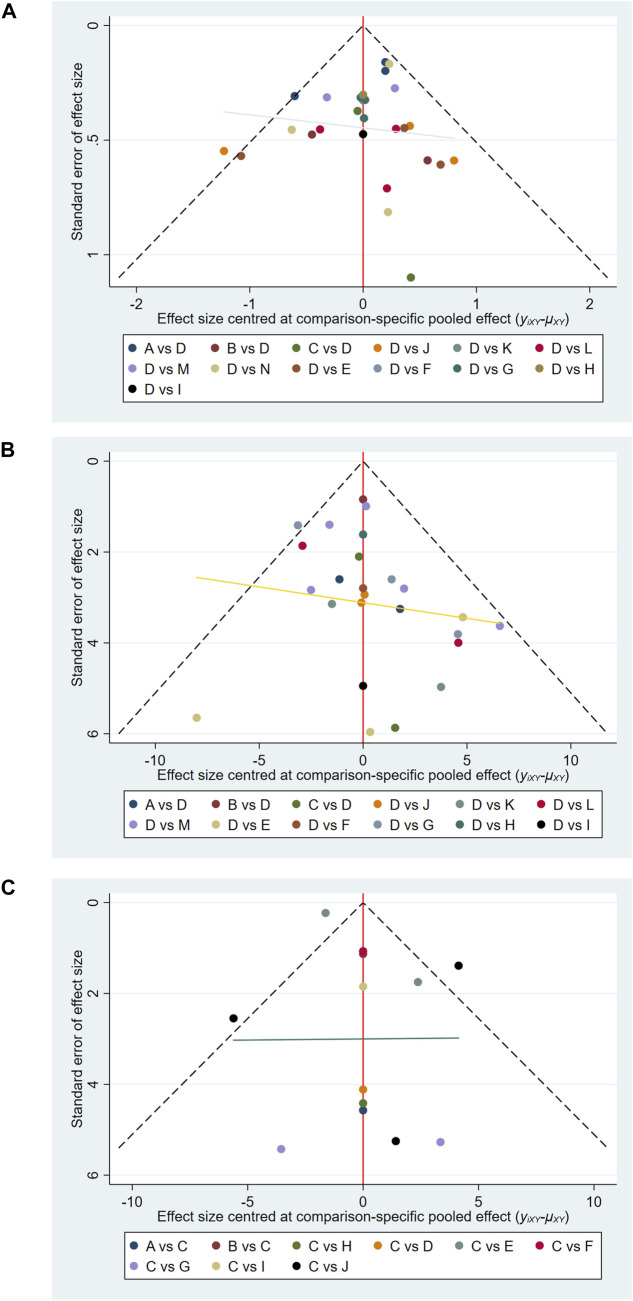
Comparison-adjusted funnel plots. **(A)** Pain outcome; **(B)** Functional outcomes; **(C)** ROM.

Comparison-adjusted funnel plots were used to evaluate potential publication bias (Albanese et al., 2021). As shown in Figures 5A–C, the funnel plots for pain, functional outcomes, and ROM appeared generally symmetric, indicating a low risk of selective reporting or small-study effects.

## 4 Discussion

The findings of this NMA provide valuable insights into the comparative effectiveness of AI-assisted rehabilitation strategies for MSDs in improving pain relief, functional outcomes, and ROM. This study synthesizes evidence from 33 RCTs, offering a robust framework to guide clinical decisions and future research in the field.

### 4.1 Summary of main findings

Pain Relief: The most effective interventions for pain relief were Therapeutic Exergaming (SUCRA = 87.6%) and Robotic Exoskeleton (SUCRA = 86.3%). These interventions involve active movement and external support, facilitating improved pain management through task-specific exercises and robotic assistance. Notably, Asynchronous Telerehabilitation and Conventional or Usual Care demonstrated lower effectiveness, highlighting the critical importance of real-time feedback and interaction in rehabilitation (Louie and Eng, 2016; Jackson et al., 2014).

Functional Outcomes: Gamified Exergaming (SUCRA = 99.6%) emerged as the top intervention for functional recovery, followed by Hybrid Physical Therapy combined with Exergaming (SUCRA = 81.2%) and Therapeutic Exergaming (SUCRA = 80.4%). The gamified approach, which incorporates exercise with engaging game elements, significantly improved patient adherence, resulting in superior functional outcomes compared to more traditional rehabilitation methods (Sánchez-Gil et al., 2025; Zhu et al., 2023).

ROM: Single-Joint Rehab Robot (SUCRA = 84.7%) and AI-Feedback Motion Training (SUCRA = 83.7%) were the most effective interventions for ROM improvement. These interventions, which focus on targeted, joint-specific rehabilitation, outperformed more general rehabilitation strategies such as Gamified Exergaming and Conventional or Usual Care (Kapil et al., 2025; Giggins et al., 2013).

### 4.2 Interpretation of Results

#### 4.2.1 Pain relief

The results of this analysis indicate that Therapeutic Exergaming and Robotic Exoskeletons are the most effective interventions for pain relief, with Therapeutic Exergaming (SUCRA = 87.6%) and Robotic Exoskeletons (SUCRA = 86.3%) ranking highest in the NMA. The underlying mechanism driving the effectiveness of these interventions likely lies in their ability to combine active rehabilitation with external support and real-time feedback. Therapeutic Exergaming, which integrates exercise and gaming elements, engages patients in task-specific movements that are not only therapeutic but also enjoyable. By involving the patient in goal-directed activities, this method enhances motivation and helps manage pain through increased physical activity, which has been shown to release endorphins and promote pain reduction (Louie and Eng, 2016; Levin et al., 2015). Additionally, the external support provided by the Robotic Exoskeletons helps alleviate the burden of movement on the patient’s joints and muscles, reducing strain and mitigating pain, especially in patients with severe impairments. Robotic exoskeletons are designed to assist with precise, controlled movements, which not only improve functional capacity but also help in pain modulation by promoting proper alignment and reducing compensatory movements that might exacerbate pain (Davenport and Kalakota, 2019; Subramanian et al., 2010).

On the other hand, Asynchronous Telerehabilitation (SUCRA = 4.2%) and Conventional or Usual Care (SUCRA = 12.0%) consistently ranked the lowest across all outcome domains. This finding is consistent with previous research suggesting that interventions that lack real-time, personalized feedback are less effective in providing pain relief. Asynchronous Telerehabilitation, which relies on pre-recorded materials and lacks synchronous interaction with healthcare providers, may fail to address the immediate needs of patients, such as correcting improper movements or adjusting therapy intensity. The absence of real-time engagement reduces the opportunity for timely adjustments, which is critical in managing pain effectively, especially in patients with chronic conditions or acute flare-ups (Topol, 2019; Koepp et al., 1998).

#### 4.2.2 Functional outcomes

The results for functional outcomes show that Gamified Exergaming (SUCRA = 99.6%) is the most effective intervention, followed by Hybrid Physical Therapy combined with Exergaming (SUCRA = 81.2%) and Therapeutic Exergaming (SUCRA = 80.4%). The superiority of Gamified Exergaming underscores the importance of patient engagement in rehabilitation. Traditional rehabilitation methods often struggle with patient adherence, especially when exercises become repetitive or monotonous. However, by integrating game mechanics, such as rewards, levels, and competitive elements, Gamified Exergaming addresses this challenge by making rehabilitation more enjoyable and motivating. The ability of game-based platforms to provide immediate, tangible rewards helps increase intrinsic motivation, which is critical for sustained participation and functional recovery (Sánchez-Gil et al., 2025; Li et al., 2021).

These findings are consistent with the concept of “gamification,” which has been shown to increase both short-term and long-term engagement in rehabilitation programs. Additionally, Gamified Exergaming often incorporates real-time feedback and progress tracking, which allows patients to visualize improvements in their functional abilities. This not only provides motivation but also reinforces the patient’s sense of accomplishment, contributing to better functional recovery (Alfieri et al., 2022; Barry et al., 2014).

In contrast, AI-Feedback Motion Training (SUCRA = 17.8%) and Conventional or Usual Care (SUCRA = 17.1%) ranked significantly lower for functional outcomes. Although AI-Feedback Motion Training provides real-time feedback on movement quality, it does not necessarily address the broader functional issues that patients with MSDs face, such as strength, endurance, or coordination. Functional recovery often requires a multifaceted approach that involves not only improving movement quality but also rebuilding strength, improving endurance, and enhancing motor control, areas in which Gamified Exergaming excels (Cottrell et al., 2017; Holden, 2005).

#### 4.2.3 ROM

In terms of ROM, Single-Joint Rehab Robot (SUCRA = 84.7%) and AI-Feedback Motion Training (SUCRA = 83.7%) were the most effective interventions. The Single-Joint Rehab Robot focuses on joint-specific rehabilitation, offering highly controlled, targeted exercises that can precisely address the limitations in ROM associated with specific musculoskeletal disorders. The precision of these robots allows for incremental increases in joint mobility without overstressing the joint, thereby promoting both recovery and pain reduction. This approach is particularly beneficial for patients with localized joint stiffness, such as those with knee or shoulder osteoarthritis, where the joint’s range of motion is severely restricted (Kapil et al., 2025; Langhorne et al., 2009).

AI-Feedback Motion Training, which involves real-time monitoring and correction of movement patterns, also demonstrated strong performance in improving ROM. The feedback provided by the AI system enables patients to adjust their movements instantly, ensuring that exercises are performed correctly and efficiently. By preventing improper movements, which can lead to further injury or discomfort, AI-Feedback Motion Training helps patients optimize their rehabilitation process, leading to greater improvements in joint mobility. Furthermore, AI-Feedback Motion Training can be personalized to suit individual needs, which may explain its effectiveness across a variety of MSDs (Huang and Krakauer, 2009; Dobkin, 2004).

In contrast, Gamified Exergaming (SUCRA = 31.2%) and Conventional or Usual Care (SUCRA = 15.0%) performed less effectively in improving ROM. While Gamified Exergaming has proven benefits in improving functional outcomes, it may not provide the specific, targeted interventions needed to address joint stiffness. As a more generalized exercise intervention, it may not be able to provide the level of specificity required for patients with significant ROM limitations. Conventional or Usual Care, which typically lacks the personalization and intensity of AI-assisted interventions, showed the lowest rankings, reinforcing the idea that more tailored, technology-driven approaches are superior for improving ROM (Cullen et al., 2012; Proffitt and Lange, 2015).

##### 4.2.3.1 Implications of findings

The findings from this study emphasize the transformative potential of AI-assisted rehabilitation interventions, particularly those that integrate real-time feedback, personalized treatment plans, and gamification elements. These technologies represent a shift from traditional rehabilitation approaches, offering a more engaging, individualized, and precise means of addressing the complex needs of patients with MSDs. The success of interventions such as Therapeutic Exergaming, Robotic Exoskeletons, and Gamified Exergaming suggests that integrating technological advancements into rehabilitation practices can significantly enhance patient outcomes (Benjamin et al., 2014; Liloia et al., 2021; Laver et al., 2020).

However, the findings also highlight that not all AI-assisted interventions are equally effective across all domains. For example, while Gamified Exergaming excels in improving functional outcomes, it may not be as effective in improving ROM, which requires more targeted, joint-specific interventions. Similarly, AI-Feedback Motion Training and Single-Joint Rehab Robot are highly effective for ROM but may not address the broader aspects of functional recovery in the same way as Gamified Exergaming (Lashkari et al., 2010; Zhang et al., 2018; Krakauer et al., 2012).

This underscores the importance of tailoring rehabilitation programs to the specific needs of individual patients. For instance, a patient with significant ROM limitations may benefit most from Single-Joint Rehab Robots or AI-Feedback Motion Training, while a patient seeking functional recovery and improved engagement may find Gamified Exergaming to be the most beneficial. Personalized rehabilitation plans that combine multiple interventions, leveraging the strengths of each technology, may provide the best outcomes for patients with MSDs (Guyatt et al., 2008; Brignardello-Petersen et al., 2018; Kwakkel et al., 2008).

Building on these findings, scenario-based guidance may help clinicians optimize intervention selection. For example, patients with pronounced ROM limitations (e.g., post-arthroplasty or joint contracture) may benefit most from Single-Joint Rehab Robots or AI-Feedback Motion Training, which deliver targeted, high-precision exercises. In contrast, patients struggling with adherence or motivation may respond better to Gamified Exergaming, where the integration of rewards and competition enhances engagement and supports functional recovery. Similarly, individuals with severe mobility impairments may require the mechanical support of Robotic Exoskeletons, while those seeking accessible, home-based options could benefit from synchronous or asynchronous telerehabilitation. Such scenario-specific recommendations underscore the potential of AI-assisted rehabilitation to provide not only effective but also personalized therapeutic strategies tailored to patient needs and clinical contexts.

Furthermore, the subgroup analyses provide preliminary evidence that age, baseline disease severity, and clinical condition may act as important moderators of treatment response. Specifically, younger and mild-to-moderate patients, as well as those in the acute postoperative stage, appeared to experience greater short-term benefits from AI-assisted rehabilitation, whereas evidence for long-term effects in older or more severe patients remains limited.

Importantly, while the present evidence demonstrates clear short-term improvements in pain, function, and ROM, it remains uncertain whether these benefits persist in the long term. Sustained rehabilitation outcomes likely depend on continuous patient engagement, integration of AI tools into daily self-management, and adherence over months or years. Only a few included RCTs extended beyond 6–12 months, and their findings suggest that early gains may attenuate without ongoing reinforcement. Thus, the translation of short-term benefits into durable functional recovery should be interpreted cautiously.

### 4.3 Limitations

While this study provides comprehensive insights, several limitations must be acknowledged. First, the included studies varied in terms of sample sizes, intervention durations, and outcome measures, which could introduce heterogeneity into the analysis. Although the NMA methodology accounts for these differences, further research with more standardized protocols is needed to enhance the reliability of the findings (Wang et al., 2022). Additionally, the majority of included studies were short-term, and thus the long-term effects of AI-assisted rehabilitation interventions remain unclear. Future studies with extended follow-up periods are essential to assess the sustainability of the observed benefits (Taylor et al., 2017). Notably, the rehabilitation protocols of included RCTs varied substantially in frequency, session duration, supervision, setting, which may have influenced the observed outcomes. Future trials should standardize and transparently report these protocol elements to facilitate cross-study comparisons.

In addition, it should be noted that the credibility assessment revealed major concerns for heterogeneity and incoherence in several comparisons. This indicates that between-study variability and potential inconsistency across direct and indirect evidence may have influenced some treatment effect estimates. Consequently, although SUCRA rankings provide a useful overview of relative performance, the confidence in these rankings is tempered by these methodological limitations. These issues highlight the need for cautious interpretation of our findings and underscore the importance of conducting further high-quality, standardized RCTs to reduce heterogeneity and improve network consistency.

Another important limitation is that most included trials reported only short-term outcomes, typically between 2 and 12 weeks. The lack of long-term follow-up data restricts our ability to determine whether the observed benefits of AI-assisted rehabilitation are sustained over time. As a result, conclusions regarding the durability and clinical relevance of these effects should be interpreted with caution. Future large-scale studies with extended follow-up periods are essential to establish the long-term efficacy, safety, and cost-effectiveness of these interventions.

### 4.4 Clinical implications

The results of this study have significant clinical implications. Therapeutic Exergaming, Robotic Exoskeletons, and Gamified Exergaming represent promising interventions that can enhance patient outcomes in terms of pain relief and functional recovery. Given their high patient engagement and potential for improving adherence, these technologies could complement traditional rehabilitation programs or even serve as standalone treatments in outpatient or home settings. Clinicians should consider incorporating these AI-assisted interventions into rehabilitation programs for individuals with MSDs, particularly in settings where traditional therapy may be limited by patient engagement or resource constraints (Topol, 2019; Louie and Eng, 2016; Sánchez-Gil et al., 2025).

In terms of clinical applicability, the feasibility and scalability of AI-assisted rehabilitation strategies should also be considered. Low-cost and highly accessible approaches such as telerehabilitation, mobile app–supported education/self-management, and exergaming are feasible in routine care and home-based settings, with evidence showing comparable effectiveness to conventional care and, in some cases, lower short-term healthcare costs (e.g., real-time telerehabilitation for musculoskeletal conditions; randomized tele-rehab trials in knee osteoarthritis; virtual in-home PT after total knee arthroplasty; postoperative education apps) (Jirasakulsuk et al., 2022; Huo et al., 2024; Prvu et al., 2020; Timmers et al., 2019). In contrast, robotics (e.g., electromechanical gait trainers, powered exoskeletons) can improve selected outcomes but typically require substantial capital investment, maintenance, trained personnel, and specialized space, which constrains widespread deployment beyond well-resourced centers (Mehrholz et al., 2020; Li et al., 2021; Charette et al., 2023; Cano-de-la-Cuerda et al., 2024; Postol et al., 2024). From a scalability perspective, mobile apps and tele-platforms are attractive because they can be delivered remotely at population scale, including to underserved regions, whereas robotics-based interventions are less scalable at present due to budget impact and implementation barriers despite growing evidence and emerging cost-effectiveness analyses in specific health-system contexts (Timmers et al., 2019; Pinto et al., 2020; Shankar et al., 2025). Addressing these practical considerations is crucial for translating current evidence into real-world rehabilitation practice.

### 4.5 Future research directions

This study highlights the need for further investigation into the long-term effects of AI-assisted rehabilitation strategies. Future research should focus on large-scale, multicenter trials that assess the sustainability of the benefits observed in this NMA. Additionally, studies exploring the combination of different AI-assisted technologies (e.g., integrating AI-Feedback Motion Training with Gamified Exergaming) may offer even more effective rehabilitation solutions (Kapil et al., 2025; Alshami et al., 2025).

Another important area for future research is the examination of AI-assisted rehabilitation in diverse patient populations, including those with different types of MSDs or comorbidities. Personalized approaches that take into account individual patient characteristics, such as severity of the condition, age, and functional status, are likely to improve the efficacy of AI-assisted interventions (Topol, 2019; Louie and Eng, 2016).

## 5 Conclusion

AI-assisted rehabilitation interventions, particularly Therapeutic Exergaming, Robotic Exoskeletons, and Gamified Exergaming, have shown significant potential in improving pain relief, functional recovery, and ROM. These technologies offer personalized, data-driven rehabilitation solutions that effectively complement traditional treatment methods. However, further research, especially long-term follow-up studies, is necessary to assess the long-term effects of these interventions and to optimize their integration into clinical practice.

## Data Availability

The original contributions presented in the study are included in the article/Supplementary Material, further inquiries can be directed to the corresponding authors.
