# Supplementary material for: Effectiveness of AI-assisted rehabilitation for musculoskeletal disorders: a network meta-analysis of pain, range of motion, and functional outcomes
Source: Front Bioeng Biotechnol. 2025 Oct 16;13:1660524. doi: 10.3389/fbioe.2025.1660524 (PMC12571919; doi:10.3389/fbioe.2025.1660524)
Supplement: Supplementary file 1 [file Supplementaryfile1.docx]

**Supplementary Table S1. Search strategy used in PubMed.**

| #1 | (“Artificial Intelligence”[MeSH Terms] OR “Machine Learning”[MeSH Terms] OR “Deep Learning”[MeSH Terms] OR “Robotics”[MeSH Terms] OR “Telerehabilitation”[MeSH Terms] OR “Virtual Reality”[MeSH Terms] OR “Video Games”[MeSH Terms] OR “Augmented Reality”[MeSH Terms] OR “AI-assisted Rehabilitation” OR “Neural Networks” OR “Cognitive Rehabilitation” OR “Intelligent Systems” OR “Adaptive Learning” OR “Human-Machine Interaction” OR “Robotic Exoskeletons”) AND “Rehabilitation”[MeSH Terms] |
| --- | --- |
| #2 | “Artificial Intelligence” OR “AI-assisted” OR “AI-supported” OR “AI-based” OR “Machine Learning” OR “Deep Learning” OR “Intelligent Feedback System” OR “Smart Rehabilitation” OR “Digital Health” OR “Digital Rehabilitation” OR “App-based Rehabilitation” OR “Mobile Health” OR “mHealth” OR “Telerehabilitation” OR “Remote Rehabilitation” OR “Virtual Reality Rehabilitation” OR “VR Rehabilitation” OR “Gamified Rehabilitation” OR “Exergaming” OR “Serious Games” OR “Robotic Rehabilitation” OR “Rehabilitation Robot” OR “Wearable Technology” OR “Motion Feedback System” OR “Telehealth” OR “Health Apps” OR “Personalized Rehabilitation” OR “Biofeedback” |
| #3 | #1 OR #2 |
| #4 | “Musculoskeletal Diseases”[MeSH Terms] OR “Musculoskeletal Pain”[MeSH Terms] OR “Low Back Pain”[MeSH Terms] OR “Neck Pain”[MeSH Terms] OR “Osteoarthritis”[MeSH Terms] OR “Tendinopathy”[MeSH Terms] OR “Arthralgia”[MeSH Terms] OR “Joint Diseases”[MeSH Terms] OR “Post-traumatic Pain” OR “Chronic Pain” OR “Muscle Strain” OR “Bone Fractures” OR “Postoperative Pain” OR “Knee Injuries” OR “Hip Disorders” OR “Spinal Disorders” |
| #5 | “Musculoskeletal Disorders” OR “Musculoskeletal Injuries” OR “MSDs” OR “Joint Disorders” OR “Osteoarthritis” OR “Back Pain” OR “Neck Pain” OR “Knee Pain” OR “Shoulder Pain” OR “Elbow Pain” OR “Ankle Injury” OR “Tendinopathy” OR “Tendon Injury” OR “Soft Tissue Injuries” OR “Ligament Injury” OR “Postoperative Rehabilitation” OR “Orthopedic Rehabilitation” OR “Sports Injuries” OR “Trauma Recovery” OR “Chronic Disease Rehabilitation” OR “Neurorehabilitation” OR “Movement Disorders” |
| #6 | #4 OR #5 |
| #7 | “Rehabilitation Robotics” OR “Robotic Exoskeletons” OR “AI-assisted Physiotherapy” OR “Interactive Rehabilitation” OR “Adaptive Rehabilitation Technology” OR “Musculoskeletal Rehabilitation” OR “Telemedicine Rehabilitation” OR “Virtual Physical Therapy” OR “Intelligent Rehabilitation Systems” |
| #8 | #3 AND #6 AND #7 |

**Supplementary Table S2. Characteristics of the randomized controlled trials included in the network meta-analysis.**

| Author (Year) | Country or Region | Study Design | Population | Sample Size (Intervention / Control) | Intervention | Comparator | Duration of Intervention | Outcomes |
| --- | --- | --- | --- | --- | --- | --- | --- | --- |
| Anan et al. (2021) | Japan | Randomized Controlled Trial | Musculoskeletal disorders | 48 / 46 | AI-Prescription App | Conventional or Usual Care | 12 weeks, 7 sessions/week, self-paced duration | Pain |
| Toelle et al. (2019) | Germany | Randomized Controlled Trial | Musculoskeletal disorders | 48 / 46 | Multimodule Digital App | Conventional or Usual Care | 12 weeks, ≥4 sessions/week, self-paced duration | Pain  Functional Outcomes |
| Cetin et al. (2022) | Turkey | Randomized Controlled Trial | Musculoskeletal disorders | 21 / 20 | Immersive VR System | Conventional or Usual Care | 6 weeks, 3 sessions/week, 40 minutes/session (20 min MC + 20 min VR) | Functional Outcomes  Range of Motion |
| Rezaei et al. (2019) | Iran | Randomized Controlled Trial | Musculoskeletal disorders | 21 / 21 | Feedback VR Platform | Conventional or Usual Care | 4 weeks, 2 sessions/week, 21 minutes/session | Pain  Functional Outcomes |
| Sarig Bahat et al. (2015) | Australia & Israel | Randomized Controlled Trial | Musculoskeletal disorders | 16 / 16 | Immersive VR System | Conventional or Usual Care | 5 weeks, 1–2 sessions/week (total 4–6 sessions), 30 minutes/session, plus continued home exercise until 3-month follow-up | Pain  Functional Outcomes  Range of Motion |
| Sarig Bahat et al. (2018) | Australia & Israel | Randomized Controlled Trial | Musculoskeletal disorders | 48/ 44 | Immersive VR System | Conventional or Usual Care | 4 weeks, 4 days/week, 4 sessions/day, 5 minutes/session, remotely supervised | Pain  Functional Outcomes  Range of Motion |
| Collado-Mateo et al. (2017) | Spain | Randomized Controlled Trial | Musculoskeletal disorders | 42 / 41 | Therapeutic Exergaming | Conventional or Usual Care | 8 weeks, 2 sessions/week, 60 minutes/session (group of 3) | Pain |
| Jin et al. (2018) | China | Randomized Controlled Trial | Musculoskeletal disorders | 33 / 33 | Immersive VR System | Conventional or Usual Care | From postoperative day 2, 2 weeks or until discharge | Pain  Functional Outcomes  Range of Motion |
| Piqueras et al. (2013) | Spain | Randomized Controlled Trial | Musculoskeletal disorders | 72 / 70 | Asynchronous Telerehab | Conventional or Usual Care | 2 weeks, 5 sessions/week, mix of supervised and home-based (total 10 sessions) | Pain  Functional Outcomes  Range of Motion |
| Yoon & Son (2020) | South Korea | Randomized Controlled Trial | Musculoskeletal disorders | 18 / 18 | Immersive VR System | Conventional or Usual Care | 2 weeks, 5 days/week, 20 minutes/day (starting week 3 post-op) | Functional Outcomes |
| Bettger et al. (2020) | USA | Randomized Controlled Trial | Musculoskeletal disorders | 143 / 144 | Feedback VR Platform | Conventional or Usual Care | 12 weeks post-op, frequency individualized | Pain  Functional Outcomes  Range of Motion |
| Kim et al. (2014) | South Korea | Randomized Controlled Trial | Musculoskeletal disorders | 15 / 15 | Gamified Exergaming | Conventional or Usual Care | 4 weeks, 3 sessions/week, 30 minutes/session | Pain  Functional Outcomes |
| Dahl-Popolizio et al. (2014) | USA | Randomized Controlled Trial | Musculoskeletal disorders | 4 / 4 | Therapeutic Exergaming | Conventional or Usual Care | 6 sessions total, outpatient clinic | Pain  Functional Outcomes  Range of Motion |
| Allen et al. (2018) | USA | Randomized Controlled Trial | Musculoskeletal disorders | 142 / 68 | Multimodule Digital App | Conventional or Usual Care | 12 months, primary endpoint at 4 months | Pain  Functional Outcomes |
| Rini et al. (2015) | USA | Randomized Controlled Trial | Musculoskeletal disorders | 58 / 55 | Multimodule Digital App | Conventional or Usual Care | 8 weeks, 1 module/week, 35–45 minutes/session, self-paced | Pain |
| Bossen et al. (2013) | Netherlands | Randomized Controlled Trial | Musculoskeletal disorders | 100 / 99 | AI-Prescription App | Conventional or Usual Care | 9 weeks, 1 module/week, self-paced with auto feedback | Pain  Functional Outcomes |
| Prabhu et al. (2020) | USA | Randomized Controlled Trial | Musculoskeletal disorders | 8 / 8 | AI-Feedback Motion Training | Conventional or Usual Care | Pre-op and post-op, 1 session each, 5 minutes/session | Pain |
| Bäcker et al. (2021) | Germany | Randomized Controlled Trial | Musculoskeletal disorders | 20 / 15 | Gamified Exergaming | Conventional or Usual Care | 6 weeks, 3–5 sessions/day, 5 minutes/session | Pain  Functional Outcomes  Range of Motion |
| Hardt et al. (2018) | Germany | Randomized Controlled Trial | Musculoskeletal disorders | 22 / 25 | Therapeutic Exergaming | Conventional or Usual Care | Immediate post-op, 3–5 sessions/day, 5 minutes/session, ~7 days total | Pain  Functional Outcomes  Range of Motion |
| Timmers et al. (2019) | Netherlands | Randomized Controlled Trial | Musculoskeletal disorders | 114 / 99 | Multimodal Digital Platform | Conventional or Usual Care | 4 weeks post-discharge | Pain  Functional Outcomes |
| Tripuraneni et al. (2021) | USA | Randomized Controlled Trial | Musculoskeletal disorders | 153 / 184 | Multimodule Digital App | Conventional or Usual Care | 2 weeks pre-op + 6 weeks post-op, guided program, continued to 12 months (self-directed) | Functional Outcomes  Range of Motion |
| Yu et al. (2023) | South Korea | Randomized Controlled Trial | Musculoskeletal disorders | 12 / 12 | AI-Feedback Motion Training | Conventional or Usual Care | 4 weeks, 3 sessions/week, 30 minutes/session | Pain  Functional Outcomes  Range of Motion |
| Maeda et al. (2024) | Japan | Randomized Controlled Trial | Musculoskeletal disorders | 40 / 36 | Single-Joint Rehab Robot | Conventional or Usual Care | 2 weeks post-op, 5 sessions/week (total 10 sessions) | Pain  Functional Outcomes  Range of Motion |
| Tanaka et al. (2017) | Japan | Randomized Controlled Trial | Musculoskeletal disorders | 13 / 13 | Robotic Exoskeleton | Conventional or Usual Care | 2 weeks, 7 sessions/week, 40 minutes/day | Pain  Functional Outcomes |
| Kotani et al. (2020) | Japan | Randomized Controlled Trial | Musculoskeletal disorders | 12 / 10 | Single-Joint Rehab Robot | Conventional or Usual Care | 3 sessions total between POD5 and POD10 | Pain  Functional Outcomes  Range of Motion |
| Azma et al. (2017) | Iran | Randomized Controlled Trial | Musculoskeletal disorders | 27 / 27 | Synchronous/Supported Telerehab | Conventional or Usual Care | 6 weeks, 3 sessions/week (total 18 sessions) | Pain  Functional Outcomes |
| Bini & Mahajan (2016) | USA | Randomized Controlled Trial | Musculoskeletal disorders | 14 / 15 | Asynchronous Telerehab | Conventional or Usual Care | 3 months, continuous remote interaction, flexible frequency | Pain  Functional Outcomes  Range of Motion |
| Ditchburn et al. (2020) | United Kingdom | Randomized Controlled Trial | Musculoskeletal disorders | 27 / 27 | Therapeutic Exergaming | Conventional or Usual Care | 6 weeks, 2 sessions/week, 40 minutes/session | Pain |
| Zadro et al. (2019) | Australia | Randomized Controlled Trial | Musculoskeletal disorders | 30 / 30 | Gamified Exergaming | Conventional or Usual Care | 8 weeks, frequency not specified | Pain  Functional Outcomes |
| Albanese et al. (2021) | Italy | Randomized Controlled Trial | Musculoskeletal disorders | 10 / 13 | Single-Joint Rehab Robot | Conventional or Usual Care | 3 weeks, 4–5 sessions/week, 90 minutes/session | Pain  Functional Outcomes  Range of Motion |
| Marcuzzi et al. (2023) | Norway | Randomized Controlled Trial | Musculoskeletal disorders | 97 / 98 | AI-Prescription App | Conventional or Usual Care | 6 months, self-paced access via app or website | Pain  Functional Outcomes |
| Zhang, Y.; Li, H.; Huang, R. (2024) | China | Randomized Controlled Trial | Musculoskeletal disorders | 14 / 16 / | AI-Feedback Motion Training | Conventional or Usual Care | 8 weeks, 3 sessions/week, ~40 minutes/session | Functional Outcomes |
| Mete & Sari (2022) | Turkey | Randomized Controlled Trial | Musculoskeletal disorders | 30 / 30 | Hybrid PT + Exergaming | Conventional or Usual Care | 6 weeks, 5 sessions/week, 20 minutes/session (Exergaming) | Pain  Functional Outcomes  Range of Motion |

***Note:*** *This table summarizes the key characteristics of 33 randomized controlled trials (RCTs) included in the network meta-analysis. All studies involved patients with musculoskeletal disorders (MSDs) and compared AI-assisted rehabilitation strategies with conventional or usual care. Interventions varied in type, duration, and frequency. Outcomes were classified into three domains: Pain, Functional Outcomes, and Range of Motion (ROM).*

**Supplementary Table S2a. Rehabilitation Protocols Comparison of Included RCTs**

| Author (Year) | Intervention Type | Frequency | Session Duration | Total Duration | Supervision | Setting | Exercise Type |
| --- | --- | --- | --- | --- | --- | --- | --- |
| Anan et al. (2021) | AI-Prescription App | 7 sessions/week | Self-paced | 12 weeks | Self-managed | Home/Remote | Exercise prescription, self-paced activities |
| Toelle et al. (2019) | Multimodule Digital App | ≥4 sessions/week | Self-paced | 12 weeks | Self-managed | Home/Remote | Digital exercise modules |
| Cetin et al. (2022) | Immersive VR System | 3 sessions/week | 40 min (20 MC + 20 VR) | 6 weeks | Therapist supervised | Clinic | Motor control + VR tasks |
| Rezaei et al. (2019) | Feedback VR Platform | 2 sessions/week | 21 min | 4 weeks | Therapist supervised | Clinic | VR-based feedback training |
| Sarig Bahat et al. (2015) | Immersive VR System | 1–2 sessions/week | 30 min | 5 weeks + home program | Therapist supervised | Clinic + Home | Neck mobility & pain exercises |
| Sarig Bahat et al. (2018) | Immersive VR System | 4 sessions/day, 4 d/w | 5 min | 4 weeks | Remote supervision | Home | High-frequency cervical training |
| Collado-Mateo et al. (2017) | Therapeutic Exergaming | 2 sessions/week | 60 min | 8 weeks | Group supervised | Clinic | Exergames for functional training |
| Jin et al. (2018) | Immersive VR System | 2 sessions/day | Varied | 2 weeks or until discharge | Therapist supervised | Hospital | Postoperative VR rehab |
| Piqueras et al. (2013) | Asynchronous Telerehab | 5 sessions/week | Variable | 2 weeks (10 sessions) | Mixed (clinic + home) | Clinic + Home | Telerehab program |
| Yoon & Son (2020) | Immersive VR System | 5 days/week | 20 min | 2 weeks (week 3 post-op) | Therapist supervised | Clinic | Knee rehabilitation |
| Bettger et al. (2020) | Feedback VR Platform | Individualized | Variable | 12 weeks post-op | Therapist guided | Clinic + Home | VR feedback rehab |
| Kim et al. (2014) | Gamified Exergaming | 3 sessions/week | 30 min | 4 weeks | Therapist supervised | Clinic | Gamified exergames |
| Dahl-Popolizio et al. (2014) | Therapeutic Exergaming | Variable | N/A | 6 sessions total | Therapist supervised | Outpatient | Exergame training |
| Allen et al. (2018) | Multimodule Digital App | 1 module/week | 35–45 min | 12 months | Self-managed | Home | App-based program |
| Rini et al. (2015) | Multimodule Digital App | 1 module/week | 35–45 min | 8 weeks | Self-managed | Home | Digital pain coping training |
| Bossen et al. (2013) | AI-Prescription App | 1 module/week | Self-paced | 9 weeks | Self-managed | Home | AI-based exercise prescription |
| Prabhu et al. (2020) | AI-Feedback Motion Training | 1 pre-op + 1 post-op | 5 min | 2 sessions total | Therapist supervised | Hospital | Biofeedback VR |
| Bäcker et al. (2021) | Gamified Exergaming | 3–5 sessions/day | 5 min | 6 weeks | Therapist supervised | Clinic | Gamified exercises |
| Hardt et al. (2018) | Therapeutic Exergaming | 3–5 sessions/day | 5 min | ~7 days (immediate post-op) | Therapist supervised | Hospital | Post-op exergames |
| Timmers et al. (2019) | Multimodal Digital Platform | Unspecified | Unspecified | 4 weeks post-discharge | Self-managed | Home | App-based education & training |
| Tripuraneni et al. (2021) | Multimodule Digital App | Guided pre+post-op | Variable | 2w pre-op + 6w post-op (12m follow-up) | Mixed supervision | Home/Remote | App-based guided rehab |
| Yu et al. (2023) | AI-Feedback Motion Training | 3 sessions/week | 30 min | 4 weeks | Therapist supervised | Clinic | AR + motion feedback training |
| Maeda et al. (2024) | Single-Joint Rehab Robot | 5 sessions/week | N/A | 2 weeks | Therapist supervised | Hospital | Robot-assisted knee rehab |
| Tanaka et al. (2017) | Robotic Exoskeleton | 7 sessions/week | 40 min | 2 weeks | Therapist supervised | Hospital | Robot-assisted gait training |
| Kotani et al. (2020) | Single-Joint Rehab Robot | 3 sessions total | N/A | Post-op days 5–10 | Therapist supervised | Hospital | Robot-assisted rehab |
| Azma et al. (2017) | Synchronous Telerehab | 3 sessions/week | N/A | 6 weeks (18 sessions) | Therapist supervised | Remote | Tele-supervised PT |
| Bini & Mahajan (2016) | Asynchronous Telerehab | Flexible | N/A | 3 months | Self-managed | Remote | Continuous remote rehab |
| Ditchburn et al. (2020) | Therapeutic Exergaming | 2 sessions/week | 40 min | 6 weeks | Therapist supervised | Clinic | Exergames |
| Zadro et al. (2019) | Gamified Exergaming | Unspecified | Unspecified | 8 weeks | Unspecified | Unspecified | Gamified exercises |
| Albanese et al. (2021) | Single-Joint Rehab Robot | 4–5 sessions/week | 90 min | 3 weeks | Therapist supervised | Clinic | Robot-assisted rehab |
| Marcuzzi et al. (2023) | AI-Prescription App | Self-paced | N/A | 6 months | Self-managed | Remote | App-based program |
| Zhang et al. (2024) | AI-Feedback Motion Training | 3 sessions/week | 40 min | 8 weeks | Therapist supervised | Clinic | AI feedback motion training |
| Mete & Sari (2022) | Hybrid PT + Exergaming | 5 sessions/week | 20 min (Exergaming) | 6 weeks | Therapist supervised | Clinic | Hybrid PT + exergames |

***Notes:*** *This table summarizes the rehabilitation protocol characteristics of the included randomized controlled trials (RCTs), complementing the general study information presented in Supplementary Table S2. Protocol details include intervention frequency, session duration, total program duration, level of supervision (e.g., therapist-supervised, remote-guided, or self-managed), setting (clinic, hospital, home, or remote), and type of exercises performed. These elements strongly influence clinical outcomes and highlight the heterogeneity of rehabilitation approaches across studies.*

**Supplementary Table S3. Summary of Network Meta-Analysis Comparisons for Pain Outcomes and Confidence Ratings**

| Comparison | Number of studies | Within-study bias | Reporting bias | Indirectness | Imprecision | Heterogeneity | Incoherence | Confidence rating |
| --- | --- | --- | --- | --- | --- | --- | --- | --- |
| AI App:Control | 3 | No concerns | Low risk | No concerns | No concerns | Major concerns | Major concerns | High |
| AI Motion:Control | 2 | Some concerns | Low risk | No concerns | No concerns | Major concerns | Major concerns | High |
| Async Tele:Control | 2 | No concerns | Low risk | No concerns | Major concerns | No concerns | Major concerns | High |
| Control:Game Exe | 3 | Some concerns | Low risk | No concerns | No concerns | No concerns | Major concerns | High |
| Control:Hybrid Exe | 1 | No concerns | Low risk | No concerns | No concerns | Major concerns | Major concerns | High |
| Control:Multi App | 3 | Some concerns | Low risk | No concerns | No concerns | Major concerns | Major concerns | High |
| Control:Multi Platform | 1 | No concerns | Low risk | No concerns | No concerns | Major concerns | Major concerns | High |
| Control:Robo Exo | 1 | No concerns | Low risk | No concerns | No concerns | No concerns | Major concerns | High |
| Control:SJ Robot | 3 | No concerns | Low risk | No concerns | No concerns | Major concerns | Major concerns | High |
| Control:Sync Tele | 1 | No concerns | Low risk | No concerns | Major concerns | No concerns | Major concerns | High |
| Control:Ther Exe | 3 | No concerns | Low risk | No concerns | No concerns | No concerns | Major concerns | High |
| Control:VR Feed | 2 | Some concerns | Low risk | No concerns | Major concerns | No concerns | Major concerns | High |
| Control:VR Immersive | 3 | Some concerns | Low risk | No concerns | No concerns | Major concerns | Major concerns | High |
| AI App:AI Motion | 0 | Some concerns | Low risk | No concerns | Major concerns | No concerns | Major concerns | High |
| AI App:Async Tele | 0 | No concerns | Low risk | No concerns | No concerns | Major concerns | Major concerns | High |
| AI App:Game Exe | 0 | No concerns | Low risk | No concerns | Major concerns | No concerns | Major concerns | High |
| AI App:Hybrid Exe | 0 | No concerns | Low risk | No concerns | Major concerns | No concerns | Major concerns | High |
| AI App:Multi App | 0 | Some concerns | Low risk | No concerns | Major concerns | No concerns | Major concerns | High |
| AI App:Multi Platform | 0 | No concerns | Low risk | No concerns | Major concerns | No concerns | Major concerns | High |
| AI App:Robo Exo | 0 | No concerns | Low risk | No concerns | Major concerns | No concerns | Major concerns | High |
| AI App:SJ Robot | 0 | No concerns | Low risk | No concerns | Major concerns | No concerns | Major concerns | High |
| AI App:Sync Tele | 0 | No concerns | Low risk | No concerns | Major concerns | No concerns | Major concerns | High |
| AI App:Ther Exe | 0 | No concerns | Low risk | No concerns | Major concerns | No concerns | Major concerns | High |
| AI App:VR Feed | 0 | No concerns | Low risk | No concerns | Major concerns | No concerns | Major concerns | High |
| AI App:VR Immersive | 0 | Some concerns | Low risk | No concerns | Major concerns | No concerns | Major concerns | High |
| AI Motion:Async Tele | 0 | Some concerns | Low risk | No concerns | No concerns | No concerns | Major concerns | High |
| AI Motion:Game Exe | 0 | Some concerns | Low risk | No concerns | Major concerns | No concerns | Major concerns | High |
| AI Motion:Hybrid Exe | 0 | Some concerns | Low risk | No concerns | Major concerns | No concerns | Major concerns | High |
| AI Motion:Multi App | 0 | Some concerns | Low risk | No concerns | Major concerns | No concerns | Major concerns | High |
| AI Motion:Multi Platform | 0 | Some concerns | Low risk | No concerns | Major concerns | No concerns | Major concerns | High |
| AI Motion:Robo Exo | 0 | Some concerns | Low risk | No concerns | Major concerns | No concerns | Major concerns | High |
| AI Motion:SJ Robot | 0 | Some concerns | Low risk | No concerns | Major concerns | No concerns | Major concerns | High |
| AI Motion:Sync Tele | 0 | Some concerns | Low risk | No concerns | No concerns | Major concerns | Major concerns | High |
| AI Motion:Ther Exe | 0 | Some concerns | Low risk | No concerns | Major concerns | No concerns | Major concerns | High |
| AI Motion:VR Feed | 0 | Some concerns | Low risk | No concerns | Major concerns | No concerns | Major concerns | High |
| AI Motion:VR Immersive | 0 | Some concerns | Low risk | No concerns | Major concerns | No concerns | Major concerns | High |
| Async Tele:Game Exe | 0 | No concerns | Low risk | No concerns | No concerns | No concerns | Major concerns | High |
| Async Tele:Hybrid Exe | 0 | No concerns | Low risk | No concerns | No concerns | Major concerns | Major concerns | High |
| Async Tele:Multi App | 0 | Some concerns | Low risk | No concerns | No concerns | Major concerns | Major concerns | High |
| Async Tele:Multi Platform | 0 | No concerns | Low risk | No concerns | No concerns | No concerns | Major concerns | High |
| Async Tele:Robo Exo | 0 | No concerns | Low risk | No concerns | No concerns | No concerns | Major concerns | High |
| Async Tele:SJ Robot | 0 | No concerns | Low risk | No concerns | No concerns | No concerns | Major concerns | High |
| Async Tele:Sync Tele | 0 | No concerns | Low risk | No concerns | Major concerns | No concerns | Major concerns | High |
| Async Tele:Ther Exe | 0 | No concerns | Low risk | No concerns | No concerns | No concerns | Major concerns | High |
| Async Tele:VR Feed | 0 | No concerns | Low risk | No concerns | Major concerns | No concerns | Major concerns | High |
| Async Tele:VR Immersive | 0 | No concerns | Low risk | No concerns | No concerns | Major concerns | Major concerns | High |
| Game Exe:Hybrid Exe | 0 | No concerns | Low risk | No concerns | Major concerns | No concerns | Major concerns | High |
| Game Exe:Multi App | 0 | Some concerns | Low risk | No concerns | Major concerns | No concerns | Major concerns | High |
| Game Exe:Multi Platform | 0 | No concerns | Low risk | No concerns | Major concerns | No concerns | Major concerns | High |
| Game Exe:Robo Exo | 0 | No concerns | Low risk | No concerns | Major concerns | No concerns | Major concerns | High |
| Game Exe:SJ Robot | 0 | No concerns | Low risk | No concerns | Major concerns | No concerns | Major concerns | High |
| Game Exe:Sync Tele | 0 | No concerns | Low risk | No concerns | No concerns | Major concerns | Major concerns | High |
| Game Exe:Ther Exe | 0 | No concerns | Low risk | No concerns | Major concerns | No concerns | Major concerns | High |
| Game Exe:VR Feed | 0 | Some concerns | Low risk | No concerns | Major concerns | No concerns | Major concerns | High |
| Game Exe:VR Immersive | 0 | Some concerns | Low risk | No concerns | Major concerns | No concerns | Major concerns | High |
| Hybrid Exe:Multi App | 0 | Some concerns | Low risk | No concerns | Major concerns | No concerns | Major concerns | High |
| Hybrid Exe:Multi Platform | 0 | No concerns | Low risk | No concerns | Major concerns | No concerns | Major concerns | High |
| Hybrid Exe:Robo Exo | 0 | No concerns | Low risk | No concerns | Major concerns | No concerns | Major concerns | High |
| Hybrid Exe:SJ Robot | 0 | No concerns | Low risk | No concerns | Major concerns | No concerns | Major concerns | High |
| Hybrid Exe:Sync Tele | 0 | No concerns | Low risk | No concerns | Major concerns | No concerns | Major concerns | High |
| Hybrid Exe:Ther Exe | 0 | No concerns | Low risk | No concerns | Major concerns | No concerns | Major concerns | High |
| Hybrid Exe:VR Feed | 0 | No concerns | Low risk | No concerns | Major concerns | No concerns | Major concerns | High |
| Hybrid Exe:VR Immersive | 0 | No concerns | Low risk | No concerns | Major concerns | No concerns | Major concerns | High |
| Multi App:Multi Platform | 0 | Some concerns | Low risk | No concerns | Major concerns | No concerns | Major concerns | High |
| Multi App:Robo Exo | 0 | Some concerns | Low risk | No concerns | Major concerns | No concerns | Major concerns | High |
| Multi App:SJ Robot | 0 | Some concerns | Low risk | No concerns | Major concerns | No concerns | Major concerns | High |
| Multi App:Sync Tele | 0 | Some concerns | Low risk | No concerns | Major concerns | No concerns | Major concerns | High |
| Multi App:Ther Exe | 0 | Some concerns | Low risk | No concerns | No concerns | Major concerns | Major concerns | High |
| Multi App:VR Feed | 0 | Some concerns | Low risk | No concerns | Major concerns | No concerns | Major concerns | High |
| Multi App:VR Immersive | 0 | Some concerns | Low risk | No concerns | Major concerns | No concerns | Major concerns | High |
| Multi Platform:Robo Exo | 0 | No concerns | Low risk | No concerns | Major concerns | No concerns | Major concerns | High |
| Multi Platform:SJ Robot | 0 | No concerns | Low risk | No concerns | Major concerns | No concerns | Major concerns | High |
| Multi Platform:Sync Tele | 0 | No concerns | Low risk | No concerns | No concerns | Major concerns | Major concerns | High |
| Multi Platform:Ther Exe | 0 | No concerns | Low risk | No concerns | Major concerns | No concerns | Major concerns | High |
| Multi Platform:VR Feed | 0 | No concerns | Low risk | No concerns | Major concerns | No concerns | Major concerns | High |
| Multi Platform:VR Immersive | 0 | No concerns | Low risk | No concerns | Major concerns | No concerns | Major concerns | High |
| Robo Exo:SJ Robot | 0 | No concerns | Low risk | No concerns | Major concerns | No concerns | Major concerns | High |
| Robo Exo:Sync Tele | 0 | No concerns | Low risk | No concerns | No concerns | No concerns | Major concerns | High |
| Robo Exo:Ther Exe | 0 | No concerns | Low risk | No concerns | Major concerns | No concerns | Major concerns | High |
| Robo Exo:VR Feed | 0 | No concerns | Low risk | No concerns | Major concerns | No concerns | Major concerns | High |
| Robo Exo:VR Immersive | 0 | No concerns | Low risk | No concerns | Major concerns | No concerns | Major concerns | High |
| SJ Robot:Sync Tele | 0 | No concerns | Low risk | No concerns | Major concerns | No concerns | Major concerns | High |
| SJ Robot:Ther Exe | 0 | No concerns | Low risk | No concerns | Major concerns | No concerns | Major concerns | High |
| SJ Robot:VR Feed | 0 | No concerns | Low risk | No concerns | Major concerns | No concerns | Major concerns | High |
| SJ Robot:VR Immersive | 0 | No concerns | Low risk | No concerns | Major concerns | No concerns | Major concerns | High |
| Sync Tele:Ther Exe | 0 | No concerns | Low risk | No concerns | No concerns | No concerns | Major concerns | High |
| Sync Tele:VR Feed | 0 | No concerns | Low risk | No concerns | Major concerns | No concerns | Major concerns | High |
| Sync Tele:VR Immersive | 0 | No concerns | Low risk | No concerns | Major concerns | No concerns | Major concerns | High |
| Ther Exe:VR Feed | 0 | No concerns | Low risk | No concerns | No concerns | Major concerns | Major concerns | High |
| Ther Exe:VR Immersive | 0 | Some concerns | Low risk | No concerns | Major concerns | No concerns | Major concerns | High |
| VR Feed:VR Immersive | 0 | Some concerns | Low risk | No concerns | Major concerns | No concerns | Major concerns | High |

**Supplementary Table S4. Summary of Network Meta-Analysis Comparisons for Range of Motion (ROM) Outcomes and Confidence Ratings**

| Comparison | Number of studies | Within-study bias | Reporting bias | Indirectness | Imprecision | Heterogeneity | Incoherence | Confidence rating |
| --- | --- | --- | --- | --- | --- | --- | --- | --- |
| AI-Motion:Control | 1 | No concerns | Low risk | No concerns | No concerns | Major concerns | Major concerns | High |
| Async Tele:Control | 1 | Some concerns | Low risk | No concerns | Major concerns | No concerns | Major concerns | High |
| Control:Game-Exe | 1 | No concerns | Low risk | No concerns | Major concerns | No concerns | Major concerns | High |
| Control:Hybrid-Exe | 2 | Some concerns | Low risk | No concerns | Major concerns | No concerns | Major concerns | High |
| Control:Multi-App | 1 | No concerns | Low risk | No concerns | Major concerns | No concerns | Major concerns | High |
| Control:SJ-Robot | 2 | Some concerns | Low risk | No concerns | No concerns | Major concerns | Major concerns | High |
| Control:Ther-Exe | 1 | No concerns | Low risk | No concerns | Major concerns | No concerns | Major concerns | High |
| Control:VR-Feed | 1 | Some concerns | Low risk | No concerns | Major concerns | No concerns | Major concerns | High |
| Control:VR-Immersive | 3 | No concerns | Low risk | No concerns | Major concerns | No concerns | Major concerns | High |
| AI-Motion:Async Tele | 0 | Some concerns | Low risk | No concerns | Major concerns | No concerns | Major concerns | High |
| AI-Motion:Game-Exe | 0 | No concerns | Low risk | No concerns | Major concerns | No concerns | Major concerns | High |
| AI-Motion:Hybrid-Exe | 0 | No concerns | Low risk | No concerns | Major concerns | No concerns | Major concerns | High |
| AI-Motion:Multi-App | 0 | No concerns | Low risk | No concerns | Major concerns | No concerns | Major concerns | High |
| AI-Motion:SJ-Robot | 0 | No concerns | Low risk | No concerns | Major concerns | No concerns | Major concerns | High |
| AI-Motion:Ther-Exe | 0 | No concerns | Low risk | No concerns | Major concerns | No concerns | Major concerns | High |
| AI-Motion:VR-Feed | 0 | Some concerns | Low risk | No concerns | Major concerns | No concerns | Major concerns | High |
| AI-Motion:VR-Immersive | 0 | No concerns | Low risk | No concerns | Major concerns | No concerns | Major concerns | High |
| Async Tele:Game-Exe | 0 | Some concerns | Low risk | No concerns | Major concerns | No concerns | Major concerns | High |
| Async Tele:Hybrid-Exe | 0 | Some concerns | Low risk | No concerns | Major concerns | No concerns | Major concerns | High |
| Async Tele:Multi-App | 0 | Some concerns | Low risk | No concerns | Major concerns | No concerns | Major concerns | High |
| Async Tele:SJ-Robot | 0 | Some concerns | Low risk | No concerns | Major concerns | No concerns | Major concerns | High |
| Async Tele:Ther-Exe | 0 | Some concerns | Low risk | No concerns | Major concerns | No concerns | Major concerns | High |
| Async Tele:VR-Feed | 0 | Some concerns | Low risk | No concerns | Major concerns | No concerns | Major concerns | High |
| Async Tele:VR-Immersive | 0 | Some concerns | Low risk | No concerns | Major concerns | No concerns | Major concerns | High |
| Game-Exe:Hybrid-Exe | 0 | No concerns | Low risk | No concerns | Major concerns | No concerns | Major concerns | High |
| Game-Exe:Multi-App | 0 | No concerns | Low risk | No concerns | Major concerns | No concerns | Major concerns | High |
| Game-Exe:SJ-Robot | 0 | No concerns | Low risk | No concerns | Major concerns | No concerns | Major concerns | High |
| Game-Exe:Ther-Exe | 0 | No concerns | Low risk | No concerns | Major concerns | No concerns | Major concerns | High |
| Game-Exe:VR-Feed | 0 | Some concerns | Low risk | No concerns | Major concerns | No concerns | Major concerns | High |
| Game-Exe:VR-Immersive | 0 | No concerns | Low risk | No concerns | Major concerns | No concerns | Major concerns | High |
| Hybrid-Exe:Multi-App | 0 | No concerns | Low risk | No concerns | Major concerns | No concerns | Major concerns | High |
| Hybrid-Exe:SJ-Robot | 0 | Some concerns | Low risk | No concerns | No concerns | Major concerns | Major concerns | High |
| Hybrid-Exe:Ther-Exe | 0 | No concerns | Low risk | No concerns | Major concerns | No concerns | Major concerns | High |
| Hybrid-Exe:VR-Feed | 0 | Some concerns | Low risk | No concerns | Major concerns | No concerns | Major concerns | High |
| Hybrid-Exe:VR-Immersive | 0 | No concerns | Low risk | No concerns | Major concerns | No concerns | Major concerns | High |
| Multi-App:SJ-Robot | 0 | No concerns | Low risk | No concerns | Major concerns | No concerns | Major concerns | High |
| Multi-App:Ther-Exe | 0 | No concerns | Low risk | No concerns | Major concerns | No concerns | Major concerns | High |
| Multi-App:VR-Feed | 0 | Some concerns | Low risk | No concerns | Major concerns | No concerns | Major concerns | High |
| Multi-App:VR-Immersive | 0 | No concerns | Low risk | No concerns | Major concerns | No concerns | Major concerns | High |
| SJ-Robot:Ther-Exe | 0 | No concerns | Low risk | No concerns | Major concerns | No concerns | Major concerns | High |
| SJ-Robot:VR-Feed | 0 | Some concerns | Low risk | No concerns | Major concerns | No concerns | Major concerns | High |
| SJ-Robot:VR-Immersive | 0 | No concerns | Low risk | No concerns | Major concerns | No concerns | Major concerns | High |
| Ther-Exe:VR-Feed | 0 | Some concerns | Low risk | No concerns | Major concerns | No concerns | Major concerns | High |
| Ther-Exe:VR-Immersive | 0 | No concerns | Low risk | No concerns | Major concerns | No concerns | Major concerns | High |
| VR-Feed:VR-Immersive | 0 | Some concerns | Low risk | No concerns | Major concerns | No concerns | Major concerns | High |

**Supplementary Table S5. Summary of Network Meta-Analysis Comparisons for Functional Outcomes and Confidence Ratings**

| Comparison | Number of studies | Within-study bias | Reporting bias | Indirectness | Imprecision | Heterogeneity | Incoherence | Confidence rating |
| --- | --- | --- | --- | --- | --- | --- | --- | --- |
| AI-App:Control | 2 | Some concerns | Low risk | No concerns | Major concerns | No concerns | Major concerns | High |
| AI-Motion:Control | 1 | No concerns | Low risk | No concerns | Major concerns | No concerns | Major concerns | High |
| Async Tele:Control | 2 | No concerns | Low risk | No concerns | Major concerns | No concerns | Major concerns | High |
| Control:Game-Exe | 3 | Some concerns | Low risk | No concerns | No concerns | No concerns | Major concerns | High |
| Control:Hybrid-Exe | 1 | Some concerns | Low risk | No concerns | No concerns | No concerns | Major concerns | High |
| Control:Multi-App | 3 | No concerns | Low risk | No concerns | Major concerns | No concerns | Major concerns | High |
| Control:Multi-Platform | 1 | No concerns | Low risk | No concerns | No concerns | Major concerns | Major concerns | High |
| Control:SJ-Robot | 1 | No concerns | Low risk | No concerns | Major concerns | No concerns | Major concerns | High |
| Control:Sync Tele | 2 | Some concerns | Low risk | No concerns | Major concerns | No concerns | Major concerns | High |
| Control:Ther-Exe | 2 | No concerns | Low risk | No concerns | No concerns | No concerns | Major concerns | High |
| Control:VR-Feed | 2 | No concerns | Low risk | No concerns | Major concerns | No concerns | Major concerns | High |
| Control:VR-Immersive | 5 | No concerns | Low risk | No concerns | No concerns | No concerns | Major concerns | High |
| AI-App:AI-Motion | 0 | No concerns | Low risk | No concerns | Major concerns | No concerns | Major concerns | High |
| AI-App:Async Tele | 0 | No concerns | Low risk | No concerns | Major concerns | No concerns | Major concerns | High |
| AI-App:Game-Exe | 0 | Some concerns | Low risk | No concerns | No concerns | No concerns | Major concerns | High |
| AI-App:Hybrid-Exe | 0 | Some concerns | Low risk | No concerns | Major concerns | No concerns | Major concerns | High |
| AI-App:Multi-App | 0 | No concerns | Low risk | No concerns | Major concerns | No concerns | Major concerns | High |
| AI-App:Multi-Platform | 0 | No concerns | Low risk | No concerns | Major concerns | No concerns | Major concerns | High |
| AI-App:SJ-Robot | 0 | No concerns | Low risk | No concerns | Major concerns | No concerns | Major concerns | High |
| AI-App:Sync Tele | 0 | Some concerns | Low risk | No concerns | Major concerns | No concerns | Major concerns | High |
| AI-App:Ther-Exe | 0 | No concerns | Low risk | No concerns | Major concerns | No concerns | Major concerns | High |
| AI-App:VR-Feed | 0 | No concerns | Low risk | No concerns | Major concerns | No concerns | Major concerns | High |
| AI-App:VR-Immersive | 0 | No concerns | Low risk | No concerns | Major concerns | No concerns | Major concerns | High |
| AI-Motion:Async Tele | 0 | No concerns | Low risk | No concerns | Major concerns | No concerns | Major concerns | High |
| AI-Motion:Game-Exe | 0 | No concerns | Low risk | No concerns | No concerns | No concerns | Major concerns | High |
| AI-Motion:Hybrid-Exe | 0 | Some concerns | Low risk | No concerns | No concerns | Major concerns | Major concerns | High |
| AI-Motion:Multi-App | 0 | No concerns | Low risk | No concerns | Major concerns | No concerns | Major concerns | High |
| AI-Motion:Multi-Platform | 0 | No concerns | Low risk | No concerns | Major concerns | No concerns | Major concerns | High |
| AI-Motion:SJ-Robot | 0 | No concerns | Low risk | No concerns | Major concerns | No concerns | Major concerns | High |
| AI-Motion:Sync Tele | 0 | Some concerns | Low risk | No concerns | Major concerns | No concerns | Major concerns | High |
| AI-Motion:Ther-Exe | 0 | No concerns | Low risk | No concerns | No concerns | Major concerns | Major concerns | High |
| AI-Motion:VR-Feed | 0 | No concerns | Low risk | No concerns | Major concerns | No concerns | Major concerns | High |
| AI-Motion:VR-Immersive | 0 | No concerns | Low risk | No concerns | No concerns | Major concerns | Major concerns | High |
| Async Tele:Game-Exe | 0 | No concerns | Low risk | No concerns | No concerns | No concerns | Major concerns | High |
| Async Tele:Hybrid-Exe | 0 | Some concerns | Low risk | No concerns | Major concerns | No concerns | Major concerns | High |
| Async Tele:Multi-App | 0 | No concerns | Low risk | No concerns | Major concerns | No concerns | Major concerns | High |
| Async Tele:Multi-Platform | 0 | No concerns | Low risk | No concerns | Major concerns | No concerns | Major concerns | High |
| Async Tele:SJ-Robot | 0 | No concerns | Low risk | No concerns | Major concerns | No concerns | Major concerns | High |
| Async Tele:Sync Tele | 0 | Some concerns | Low risk | No concerns | Major concerns | No concerns | Major concerns | High |
| Async Tele:Ther-Exe | 0 | No concerns | Low risk | No concerns | Major concerns | No concerns | Major concerns | High |
| Async Tele:VR-Feed | 0 | No concerns | Low risk | No concerns | Major concerns | No concerns | Major concerns | High |
| Async Tele:VR-Immersive | 0 | No concerns | Low risk | No concerns | Major concerns | No concerns | Major concerns | High |
| Game-Exe:Hybrid-Exe | 0 | Some concerns | Low risk | No concerns | No concerns | Major concerns | Major concerns | High |
| Game-Exe:Multi-App | 0 | No concerns | Low risk | No concerns | No concerns | No concerns | Major concerns | High |
| Game-Exe:Multi-Platform | 0 | No concerns | Low risk | No concerns | No concerns | No concerns | Major concerns | High |
| Game-Exe:SJ-Robot | 0 | No concerns | Low risk | No concerns | No concerns | No concerns | Major concerns | High |
| Game-Exe:Sync Tele | 0 | Some concerns | Low risk | No concerns | No concerns | No concerns | Major concerns | High |
| Game-Exe:Ther-Exe | 0 | No concerns | Low risk | No concerns | No concerns | Major concerns | Major concerns | High |
| Game-Exe:VR-Feed | 0 | No concerns | Low risk | No concerns | No concerns | No concerns | Major concerns | High |
| Game-Exe:VR-Immersive | 0 | No concerns | Low risk | No concerns | No concerns | No concerns | Major concerns | High |
| Hybrid-Exe:Multi-App | 0 | Some concerns | Low risk | No concerns | No concerns | No concerns | Major concerns | High |
| Hybrid-Exe:Multi-Platform | 0 | Some concerns | Low risk | No concerns | Major concerns | No concerns | Major concerns | High |
| Hybrid-Exe:SJ-Robot | 0 | Some concerns | Low risk | No concerns | Major concerns | No concerns | Major concerns | High |
| Hybrid-Exe:Sync Tele | 0 | Some concerns | Low risk | No concerns | Major concerns | No concerns | Major concerns | High |
| Hybrid-Exe:Ther-Exe | 0 | Some concerns | Low risk | No concerns | Major concerns | No concerns | Major concerns | High |
| Hybrid-Exe:VR-Feed | 0 | Some concerns | Low risk | No concerns | Major concerns | No concerns | Major concerns | High |
| Hybrid-Exe:VR-Immersive | 0 | Some concerns | Low risk | No concerns | Major concerns | No concerns | Major concerns | High |
| Multi-App:Multi-Platform | 0 | No concerns | Low risk | No concerns | Major concerns | No concerns | Major concerns | High |
| Multi-App:SJ-Robot | 0 | No concerns | Low risk | No concerns | Major concerns | No concerns | Major concerns | High |
| Multi-App:Sync Tele | 0 | Some concerns | Low risk | No concerns | Major concerns | No concerns | Major concerns | High |
| Multi-App:Ther-Exe | 0 | No concerns | Low risk | No concerns | No concerns | No concerns | Major concerns | High |
| Multi-App:VR-Feed | 0 | No concerns | Low risk | No concerns | Major concerns | No concerns | Major concerns | High |
| Multi-App:VR-Immersive | 0 | No concerns | Low risk | No concerns | No concerns | Major concerns | Major concerns | High |
| Multi-Platform:SJ-Robot | 0 | No concerns | Low risk | No concerns | Major concerns | No concerns | Major concerns | High |
| Multi-Platform:Sync Tele | 0 | Some concerns | Low risk | No concerns | Major concerns | No concerns | Major concerns | High |
| Multi-Platform:Ther-Exe | 0 | No concerns | Low risk | No concerns | Major concerns | No concerns | Major concerns | High |
| Multi-Platform:VR-Feed | 0 | No concerns | Low risk | No concerns | Major concerns | No concerns | Major concerns | High |
| Multi-Platform:VR-Immersive | 0 | No concerns | Low risk | No concerns | Major concerns | No concerns | Major concerns | High |
| SJ-Robot:Sync Tele | 0 | Some concerns | Low risk | No concerns | Major concerns | No concerns | Major concerns | High |
| SJ-Robot:Ther-Exe | 0 | No concerns | Low risk | No concerns | Major concerns | No concerns | Major concerns | High |
| SJ-Robot:VR-Feed | 0 | No concerns | Low risk | No concerns | Major concerns | No concerns | Major concerns | High |
| SJ-Robot:VR-Immersive | 0 | No concerns | Low risk | No concerns | Major concerns | No concerns | Major concerns | High |
| Sync Tele:Ther-Exe | 0 | Some concerns | Low risk | No concerns | Major concerns | No concerns | Major concerns | High |
| Sync Tele:VR-Feed | 0 | Some concerns | Low risk | No concerns | Major concerns | No concerns | Major concerns | High |
| Sync Tele:VR-Immersive | 0 | Some concerns | Low risk | No concerns | Major concerns | No concerns | Major concerns | High |
| Ther-Exe:VR-Feed | 0 | No concerns | Low risk | No concerns | Major concerns | No concerns | Major concerns | High |
| Ther-Exe:VR-Immersive | 0 | No concerns | Low risk | No concerns | Major concerns | No concerns | Major concerns | High |
| VR-Feed:VR-Immersive | 0 | No concerns | Low risk | No concerns | Major concerns | No concerns | Major concerns | High |

**Supplementary Table S6. Classification of AI-Assisted Rehabilitation Strategies and Abbreviations**

| **Original Name** | **Abbreviation** |
| --- | --- |
| Conventional or Usual Care | Control |
| AI-Feedback Motion Training | AI-Motion |
| AI-Prescription App | AI-App |
| Asynchronous Telerehab | Async Tele |
| Synchronous/Supported Telerehab | Sync Tele |
| Gamified Exergaming | Game-Exe |
| Therapeutic Exergaming | Ther-Exe |
| Feedback VR Platform | VR-Feed |
| Immersive VR System | VR-Immersive |
| Multimodule Digital App | Multi-App |
| Multimodal Digital Platform | Multi-Platform |
| Robotic Exoskeleton | Robo-Exo |
| Single-Joint Rehab Robot | SJ-Robot |
| Hybrid Physical Therapy combined with Exergaming | Hybrid-Exe |

**Supplementary Table S7. Node-splitting analysis for assessing inconsistency in pain outcome**

| Side | Direct_Coef | Direct_SE | Indirect_Coef | Indirect_SE | Difference_Coef | Difference_SE | P | Tau |
| --- | --- | --- | --- | --- | --- | --- | --- | --- |
| A-D | 0.7902535 | 0.2410088 | 0.0495793 | 63.39207 | 0.7406741 | 63.39248 | 0.991 | 0.3496231 |
| B-D | 1.115131 | 0.4475666 | 0.4654303 | 446.0741 | 0.6497009 | 446.0746 | 0.999 | 0.3496237 |
| C-D | -0.4928768 | 0.4678789 | 2.073382 | 447.638 | -2.566258 | 447.6386 | 0.995 | 0.3496236 |
| D-E | -1.26886 | 0.3681792 | -2.84938 | 366.123 | 1.58052 | 366.1229 | 0.997 | 0.3496236 |
| D-F | -1 | 0.4758186 | -2.580232 | 626.7704 | 1.580232 | 626.7704 | 0.998 | 0.3496237 |
| D-G | -0.7173953 | 0.2837636 | -2.301202 | 377.9727 | 1.583806 | 377.9727 | 0.997 | 0.3496237 |
| D-H | -1.14 | 0.4621491 | -2.721628 | 633.6869 | 1.581628 | 633.687 | 0.998 | 0.3496237 |
| D-I | -1.7 | 0.5889415 | -3.280391 | 628.196 | 1.580391 | 628.1959 | 0.998 | 0.3496236 |
| D-J | -0.9874618 | 0.3608742 | -2.565539 | 369.3671 | 1.578077 | 369.3671 | 0.997 | 0.3496236 |
| D-K | 0.1500001 | 0.4784384 | -1.430483 | 637.0787 | 1.580483 | 637.0787 | 0.998 | 0.3496236 |
| D-L | -1.611373 | 0.3599749 | -3.191768 | 367.3079 | 1.580395 | 367.3079 | 0.997 | 0.3496241 |
| D-M | -0.5833278 | 0.3227107 | -2.162381 | 449.5171 | 1.579053 | 449.5171 | 0.997 | 0.3496238 |
| D-N | -0.7895185 | 0.3071166 | -2.371997 | 364.0524 | 1.582478 | 364.0524 | 0.997 | 0.3496237 |

***Notes:*** *A = AI-App; B = AI-Motion; C = Async Tele; D = Control; E = Game-Exe; F = Hybrid-Exe; G = Multi-App; H = Multi-Platform; I = Robo-Exo; J = SJ-Robot; K = Sync Tele; L = Ther-Exe; M = VR-Feed; N = VR-Immersive.*

**Supplementary Table S8. Node-splitting analysis for assessing inconsistency in functional outcomes**

| Side | Direct_Coef | Direct_SE | Indirect_Coef | Indirect_SE | Difference_Coef | Difference_SE | P | Tau |
| --- | --- | --- | --- | --- | --- | --- | --- | --- |
| A-D | -3.782513 | 2.366437 | -0.1501128 | 65.93851 | -3.6324 | 65.981 | 0.956 | 1.681898 |
| B-D | 0.1409989 | 1.880461 | -7.697027 | 632.1312 | 7.838026 | 632.1351 | 0.99 | 1.681641 |
| C-D | -1.796938 | 2.465481 | -5.759226 | 447.2596 | 3.962288 | 447.2767 | 0.993 | 1.681677 |
| D-E | 17.52412 | 2.875719 | 25.08086 | 365.2023 | -7.556742 | 365.1934 | 0.983 | 1.681656 |
| D-F | 8.800003 | 3.26303 | 16.35616 | 632.524 | -7.556155 | 632.5201 | 0.99 | 1.681641 |
| D-G | -0.2241915 | 1.841669 | 7.332735 | 365.2835 | -7.556927 | 365.2818 | 0.983 | 1.681665 |
| D-H | 5.470001 | 2.330616 | 13.02623 | 632.4611 | -7.556232 | 632.4612 | 0.99 | 1.681642 |
| D-I | 2.799995 | 5.224249 | 10.35616 | 632.4829 | -7.556163 | 632.4658 | 0.99 | 1.681643 |
| D-J | 1.218373 | 2.446403 | 8.774947 | 447.2032 | -7.556575 | 447.1997 | 0.987 | 1.681653 |
| D-K | 8.408121 | 2.956541 | 15.9647 | 447.2234 | -7.556583 | 447.2169 | 0.987 | 1.681654 |
| D-L | 3.68202 | 2.275483 | 11.23859 | 447.3063 | -7.556567 | 447.304 | 0.987 | 1.681652 |
| D-M | 4.897765 | 1.222481 | 12.4556 | 283.078 | -7.557837 | 283.0776 | 0.979 | 1.681684 |

***Notes:*** *A = AI-App; B = AI-Motion; C = Async Tele; D = Control; E = Game-Exe; F = Hybrid-Exe; G = Multi-App; H = Multi-Platform; I = SJ-Robot; J = Sync Tele; K = Ther-Exe; L = VR-Feed; M = VR-Immersive.*

**Supplementary Table S9. Node-splitting analysis for assessing inconsistency in range of motion (ROM) outcome**

| Side | Direct_Coef | Direct_SE | Indirect_Coef | Indirect_SE | Difference_Coef | Difference_SE | P | Tau |
| --- | --- | --- | --- | --- | --- | --- | --- | --- |
| A-C | -13.09 | 6.043308 | -0.201669 | 91.29681 | -12.88833 | 91.49661 | 0.888 | 3.952629 |
| B-C | -2.419998 | 4.109317 | -23.65525 | 632.6575 | 21.23525 | 632.6729 | 0.973 | 3.951029 |
| C-D | 6.279999 | 5.703184 | 32.35705 | 632.6321 | -26.07705 | 632.6311 | 0.967 | 3.951028 |
| C-E | 1.824877 | 2.9227 | 27.91141 | 447.3478 | -26.08653 | 447.3558 | 0.953 | 3.95117 |
| C-F | 2.200005 | 4.093269 | 28.27706 | 632.5961 | -26.07705 | 632.6075 | 0.967 | 3.951028 |
| C-G | 12.61368 | 4.701352 | 38.70022 | 447.2705 | -26.08653 | 447.2632 | 0.953 | 3.951171 |
| C-H | 11 | 5.925878 | 37.07705 | 632.6279 | -26.07705 | 632.6249 | 0.967 | 3.951022 |
| C-I | 3.099998 | 4.361811 | 29.17705 | 632.5544 | -26.07705 | 632.5641 | 0.967 | 3.951028 |
| C-J | 3.860429 | 2.834632 | 29.95645 | 365.3812 | -26.09602 | 365.3844 | 0.943 | 3.951315 |

***Notes:*** *A = AI-Motion; B = Async Tele; C = Control; D = Game-Exe; E = Hybrid-Exe; F = Multi-App; G = SJ-Robot; H = Ther-Exe; I = VR-Feed; J = VR-Immersive.*

**Supplementary Table S10. Subgroup analyses of AI-assisted rehabilitation effects on pain, functional outcomes, and range of motion across age, disease severity, and clinical condition**

| Outcome Indicator | Subgroup | Number of Studies | I² (%) | P-value (heterogeneity) | Z-value | SMD | 95% CI for SMD | P-value (effect) |
| --- | --- | --- | --- | --- | --- | --- | --- | --- |
| Pain | Age <60 | 10 | 38 | 0.07 | 2.15 | 0.40 | –0.72, –0.08 | 0.03 |
|  | Age ≥60 | 7 | 55 | 0.05 | 1.25 | 0.25 | –0.58, 0.10 | 0.11 |
|  | Mild–Moderate severity | 6 | 42 | 0.09 | 2.30 | 0.35 | –0.65, –0.05 | 0.02 |
|  | Moderate–Severe severity | 4 | 60 | 0.04 | 1.50 | 0.20 | –0.55, 0.15 | 0.13 |
|  | Acute | 5 | 30 | 0.20 | 1.85 | 0.33 | –0.70, 0.04 | 0.07 |
|  | Chronic | 12 | 65 | 0.03 | 2.60 | 0.50 | –0.82, –0.18 | 0.009 |
| Range of Motion (ROM) | Age <60 | 4 | 32 | 0.15 | 2.05 | 5.5 | 1.2, 9.8 | 0.04 |
|  | Age ≥60 | 4 | 48 | 0.07 | 1.90 | 4.8 | 0.5, 9.1 | 0.05 |
|  | Mild–Moderate severity | 3 | 38 | 0.10 | 2.10 | 6.0 | 1.0, 11.0 | 0.03 |
|  | Moderate–Severe severity | 3 | 55 | 0.05 | 1.40 | 3.5 | –0.5, 7.5 | 0.15 |
|  | Acute | 4 | 25 | 0.22 | 2.40 | 6.2 | 0.8, 11.6 | 0.02 |
|  | Chronic | 5 | 60 | 0.04 | 2.30 | 7.1 | 2.0, 12.2 | 0.02 |
| Functional Outcomes | Age <60 | 9 | 40 | 0.08 | 2.40 | 3.20 | 0.90, 5.50 | 0.02 |
|  | Age ≥60 | 7 | 52 | 0.06 | 1.70 | 2.10 | –0.20, 4.40 | 0.09 |
|  | Mild–Moderate severity | 5 | 35 | 0.12 | 2.20 | 4.00 | 1.00, 7.00 | 0.03 |
|  | Moderate–Severe severity | 4 | 58 | 0.04 | 1.60 | 2.50 | –0.10, 5.10 | 0.11 |
|  | Acute | 6 | 28 | 0.19 | 2.50 | 5.50 | 1.50, 9.50 | 0.01 |
|  | Chronic | 10 | 60 | 0.03 | 2.10 | 3.00 | 0.40, 5.60 | 0.04 |

***Notes:*** *Age subgroups were defined as* <*60 years and ≥60 years according to the mean age of participants reported in the original trials. Disease severity was classified based on baseline descriptions as mild–moderate vs. moderate–severe musculoskeletal disorders. “Acute condition” mainly refers to postoperative rehabilitation populations (e.g., total knee arthroplasty), while “chronic condition” refers to long-term musculoskeletal pain disorders, including chronic low back pain, chronic neck pain, knee osteoarthritis, and fibromyalgia. I² represents the degree of heterogeneity; P-value (heterogeneity) indicates the significance of heterogeneity; Z-value represents the test statistic for overall effect; SMD denotes standardized mean difference; 95% CI indicates the 95% confidence interval; and P-value (effect) refers to the significance of the treatment effect*

**Supplementary Table 11. SUCRA-based ranking of 13 AI-assisted rehabilitation interventions for pain, functional outcomes, and ROM, based on network meta-analysis results.**

| Outcome Indicator | Treatment | SUCRA | PrBest | MeanRank |
| --- | --- | --- | --- | --- |
| Pain | Ther-Exe | 87.6 | 29.3 | 2.6 |
|  | Robo-Exo | 86.3 | 43.5 | 2.8 |
|  | Game-Exe | 73.7 | 7.6 | 4.4 |
|  | Multi-Platform | 66.7 | 7.4 | 5.3 |
|  | AI-Motion | 65.3 | 5.8 | 5.5 |
|  | Hybrid-Exe | 59.4 | 4.4 | 6.3 |
|  | SJ-Robot | 59.0 | 1.7 | 6.3 |
|  | VR-Immersive | 47.8 | 0.2 | 7.8 |
|  | AI-App | 47.0 | 0.1 | 7.9 |
|  | Multi-App | 43.2 | 0.1 | 8.4 |
|  | VR-Feed | 36.5 | 0 | 9.3 |
|  | Control | 12.0 | 0 | 12.4 |
|  | Sync Tele | 11.3 | 0 | 12.5 |
|  | Async Tele | 4.2 | 0 | 13.5 |
| Range of Motion (ROM) | SJ-Robot | 84.7 | 31.5 | 2.4 |
|  | AI-Motion | 83.7 | 37.8 | 2.5 |
|  | Ther-Exe | 76.8 | 23.3 | 3.1 |
|  | Game-Exe | 55.7 | 5.9 | 5.0 |
|  | VR-Immersive | 44.7 | 0.2 | 6.0 |
|  | VR-Feed | 39.4 | 0.8 | 6.4 |
|  | Async Tele | 35.3 | 0.3 | 6.8 |
|  | Multi-App | 33.5 | 0.3 | 7.0 |
|  | Hybrid-Exe | 31.2 | 0.1 | 7.2 |
|  | Control | 15.0 | 0 | 8.6 |
| Functional Outcomes | Game-Exe | 99.6 | 95.9 | 1.0 |
|  | Hybrid-Exe | 81.2 | 2.2 | 3.3 |
|  | Ther-Exe | 80.4 | 1.2 | 3.4 |
|  | Multi-Platform | 64.9 | 0 | 5.2 |
|  | VR-Immersive | 62.1 | 0 | 5.6 |
|  | AI-App | 51.7 | 0 | 6.8 |
|  | VR-Feed | 50.7 | 0 | 6.9 |
|  | SJ-Robot | 43.2 | 0.6 | 7.8 |
|  | Async Tele | 35.0 | 0 | 8.8 |
|  | Sync Tele | 29.7 | 0 | 9.4 |
|  | AI-Motion | 17.8 | 0 | 10.9 |
|  | Control | 17.1 | 0 | 11.0 |
|  | Multi-App | 16.7 | 0 | 11.0 |

***Notes:*** *This table summarizes the SUCRA values, probability of being the best treatment (PrBest), and mean ranking (MeanRank) for all interventions across three outcome domains (pain, functional outcomes, and range of motion). Higher SUCRA values indicate more favorable relative rankings. Abbreviations: Conventional or Usual Care (Control); AI-Feedback Motion Training (AI-Motion); AI-Prescription App (AI-App); Asynchronous Telerehabilitation (Async Tele); Synchronous/Supported Telerehabilitation (Sync Tele); Gamified Exergaming (Game-Exe); Therapeutic Exergaming (Ther-Exe); Feedback VR Platform (VR-Feed); Immersive VR System (VR-Immersive); Multimodule Digital App (Multi-App); Multimodal Digital Platform (Multi-Platform); Robotic Exoskeleton (Robo-Exo); Single-Joint Rehabilitation Robot (SJ-Robot); Hybrid Physical Therapy combined with Exergaming (Hybrid-Exe).*


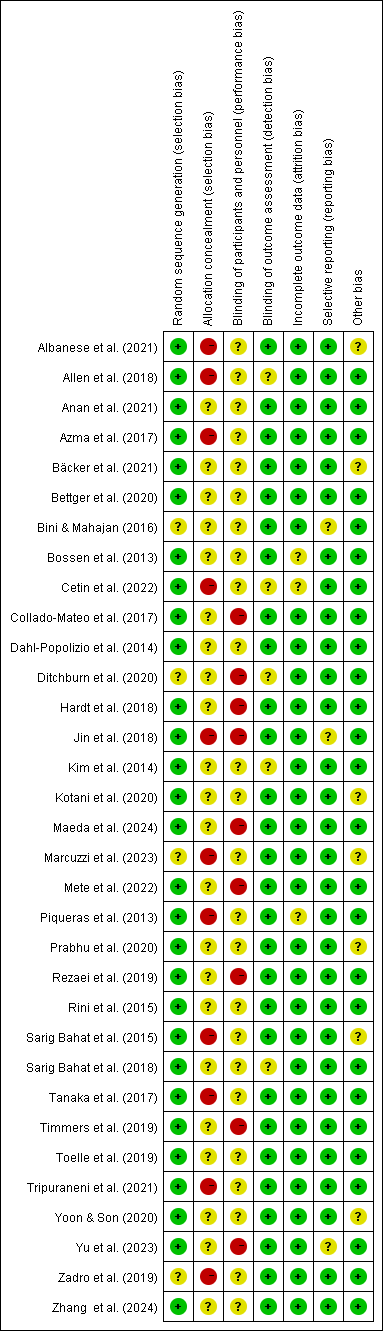


**Supplementary Figure S1. Summary of risk of bias assessments and traffic light plot generated using RevMan version 5.4.**

***Note:*** *Control = Conventional or Usual Care.*

**Supplementary Figure S2. League table comparing relative treatment effects for pain outcome**

***Note:*** *Control = Conventional or Usual Care.*

**Supplementary Figure S3. League table comparing relative treatment effects for functional outcomes**

***Note:*** *Control = Conventional or Usual Care.*

**Supplementary Figure S4. League table comparing relative treatment effects for range of motion (ROM) outcome**
